# Mapping relationships among gross motor skills in 16,989 children using network analysis

**DOI:** 10.1038/s41598-025-95924-9

**Published:** 2025-04-04

**Authors:** Roberto Vagnetti, Simon Cooper, Fabio Carlevaro, Ruth Boat, Francesca Magno, Giovanni Musella, Daniele Magistro

**Affiliations:** 1 Department of Sport Science, School of Science and Technology, Nottingham Trent University, College Drive, Clifton, Nottingham, NG11 8NS UK; 2 Polo Universitario Asti Studi Superiori (Uni-Astiss), Area Fabrizio De Andrè, 14100 Asti, Italy; 3 Dipartimento di Scienze della Vita e Biologia dei Sistemi, University of Torino, Torino, Italy; 4 School of Environment, Education and Development, University of Manchester, Manchester, M13 9PL UK

**Keywords:** Gross motor skills, Motor development, Children, Network analysis

## Abstract

The development of gross motor skills during childhood is crucial for shaping more complex movements and laying the groundwork for physical activity, and subsequently lifelong health and enhanced well-being. Performance in motor skills improves throughout development, with the greatest improvements occurring during childhood. Understanding the relationships between developing gross motor skills is essential for informing educational and intervention practices. A total of 16,989 children aged 3–11 years underwent assessment of gross motor skills. Using network analysis, gross motor skills networks were constructed for the entire sample, and stratified by age and sex. The accuracy and stability of the networks were assessed, and centrality and bridge statistics were estimated for each node. The results indicated that running and two-hand catching exhibited higher centrality and bridge statistics compared to the other nodes in the all-sample network. Additionally, it was observed that the strength between nodes decreased and their distance increased with age. These results highlight the importance of specific gross motor skills due to their significant role in relation to other skills within the network. Gross motor skills progress towards increased independence and specialisation during development, indicating the importance of early educational interventions where children could benefit from educational practices focused on catching and running.

**Supplementary Information:**

The online version contains supplementary material available at 10.1038/s41598-025-95924-9.

## Introduction

Early childhood is an essential period for the development of gross motor skills^1^. These gross motor skills involve acquiring control over and effectively using large muscle groups to produce controlled movements^2^. In essence, gross motor skills encompass full-body movements and they form movement patterns designed to achieve specific aims^3,4^. These skills encompass a wide range of movement patterns, which can be broadly categorized into locomotor skills (e.g., running, hopping, jumping etc.) and ball/object control skills (e.g., throwing, catching, and kicking a ball). The acquisition of these skills holds significance for various aspects of daily life. For example, those young people with higher levels of gross motor skills are more likely to be physically active^5^, not least due to greater (perceived) competence for many physical activities. Subsequently, gross motor skills are also important for a range of positive outcomes, including: fostering and sustaining physical fitness^6^, preventing children’s obesity^7^, promoting lifelong health^8^, nurturing social and emotional well-being^9^, enhancing adaptive skills^10^, enhancing cognitive abilities^11–13^ and influencing academic achievements^14,15^. The development of these gross motor skills is also crucial since they underpin the development of more complex movements^5,16^ and the development of activity and sport-specific abilities.

Given the importance of gross motor skill development in young people, investigating the relationships between the development of different gross motor skills is vital. Furthermore, understanding how the relationships may change over time can further shed light on the high variability that children exhibit during the development of gross motor skills^17^, and how this development may be important for motor-related problems, such as developmental coordination disorder^18,19^. Indeed, during development many factors such as expertise, body changes due to growth, and biomechanical changes affect children’s motor skills^20^. This is important to understand, given that it is hypothesised that gross motor skills follow a developmental sequence progressing from simple to more complex skills, with aspects of gross motor skills (e.g., arm swing during running) underpinning the development of more ‘complex’ skills (e.g., skipping)^20^.

It is known that the performance of motor skills increases during development and as a result of ageing^21^. Specifically, evidence suggests that the greatest improvements are achieved during childhood^22^, and this development consistently has a major role in determining motor competence^23^. The development of motor skills with ageing is influenced by various factors, including body proportions, maturation, and the availability of practice opportunities^24,25^. Roughly 75% of pre-school children can proficiently master the skill of running, while many of the other abilities such as galloping, hopping, jumping, striking, catching, kicking, and throwing are still in the process of development^1^. By the age of 7, it is anticipated that these skills will have reached an adequate level of competency, coinciding with the point at which children typically begin to participate in more specific physical activities^8^. Alongside age, it is also important to consider how sex influences the development of motor skills. Whist it has been suggested that males are more proficient in ball skills and females more proficient in locomotor skills^26–28^, a recent meta-analysis indicated that males and females have similar competence for locomotor skills, while males tend to display greater object manipulation skills^25^. It has been hypothesised that these differences in performance may be due to the different kind of activities that males and females typically participate in^25^. However, to date, how age and sex affect the relationships between gross motor skills has not been examined.

Therefore, the aim of the present study was to analyse the relationships and interactions between gross motor skills in pre-school and school aged-children. Specifically, this study aimed to: i) understand which gross motor skills act as a central component in the relationships between gross motor skills; ii) understand how relationships among gross motor skills change during development; and iii) understand whether the interactions between gross motor skills are different between boys and girls.

## Methods

### Procedure

The study was conducted in accordance with the Declaration of Helsinki and following ethical approval (Ethical committee—Torino University—ID100949), 16,989 children (51% female) aged 3–11 years (7.26 ± 2.19 years) from 122 pre-schools and primary schools in the north of Italy participated in the study. A written informed consent form was completed by parents/guardians, and verbal assent was gained from participating children. Gross motor skills were assessed with the Test of Gross Motor Development—Third Edition (TGDM-3)^29–31^. The TGMD-3 was administered in schools (typically in a gym/sports hall) during normal school hours. The TGMD-3 assesses two gross motor skill dimensions according to its two subscales: the *Locomotor skills (Ls) subscale* (run, gallop, hop, horizontal jump, skip, slide) and the *Ball skills (Bs) subscale* (one hand forehand strike of self-bounced tennis ball, kick a stationary ball, overhand throw, underhand throw, two hand strike of a stationary ball, one hand stationary dribble, two hand catch). During the assessment, each skill is observed and evaluated using qualitative performance criteria (3–5 performance criteria per skill). For each criterion, a score of 1 is awarded if it is fulfilled, and a score of 0 if it is not. The TGMD-3 was administered in accordance with the original authors’ recommendations^31^ and took approximately 20 min per child. Each skill was evaluated twice within the same testing session, immediately following the practice trial. There was no extended time lag between the two formal trials, as they were conducted consecutively to maintain consistency in evaluation and minimise potential external influences on performance. Each child’s performance was observed and scored in real time by two independent testers, who were randomly paired for each assessment session. The agreement between the scores recorded by the two testers exceeded 95%. To assess inter-rater agreement for all TGMD-3 criteria, Cohen’s kappa^32^ was calculated, with values ranging from 0.8 to 1, indicating excellent to almost perfect agreement. Additionally, inter-rater reliability for the final TGMD-3 score was evaluated using a two-way random intraclass correlation (absolute agreement), with coefficients above 0.90, signifying excellent reliability. The locomotor subscale has a maximum score of 46, and the ball skill subscale a maximum score of 54, yielding a total score out of 100. The TGMD-3 has been shown to have strong construct validity and reliability in measuring gross motor skills in children^30^.

### Preliminary analysis

**Fig. 1:**
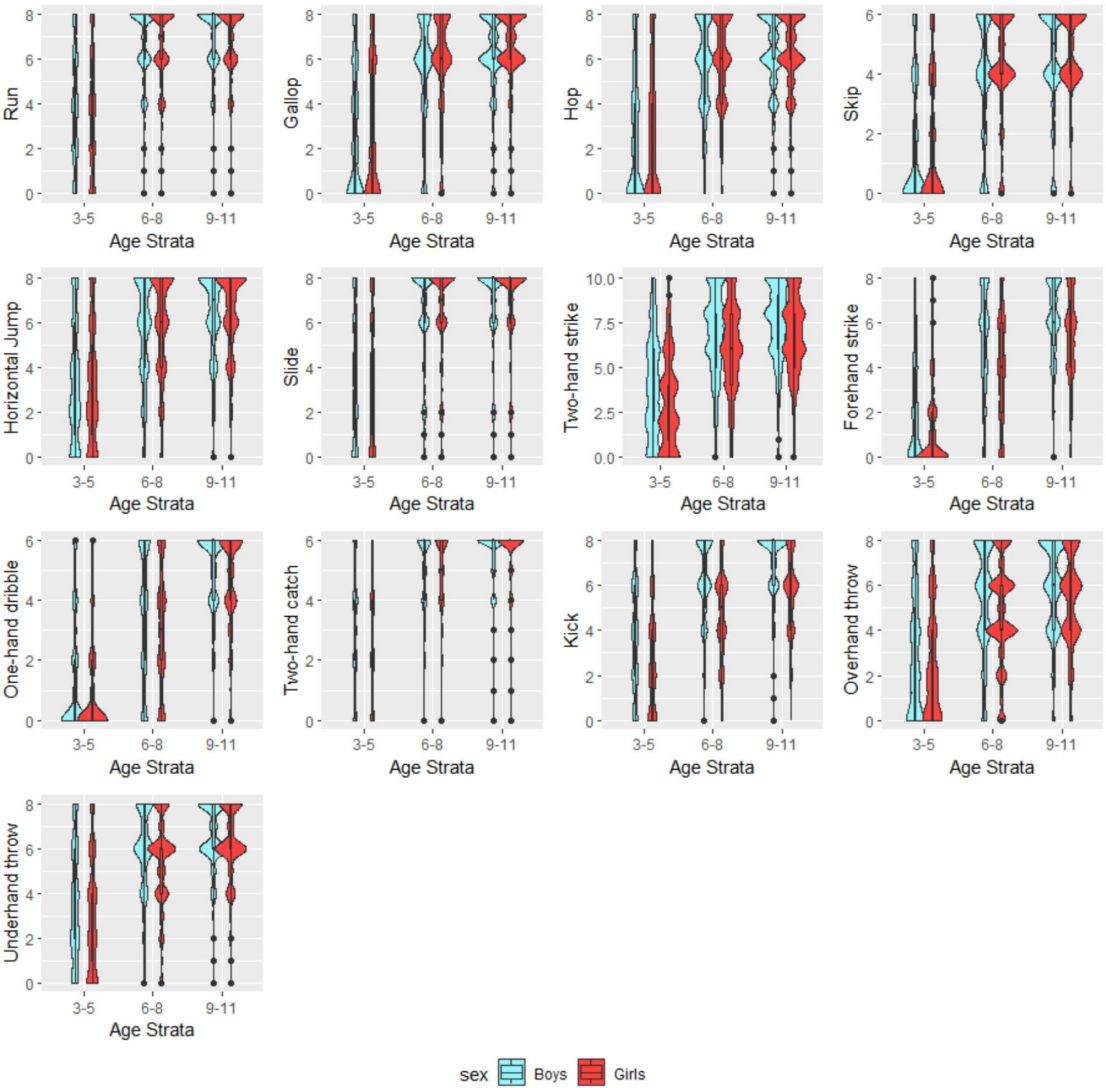
Violin plots of samples according to age strata and sex for each TGMD-3 subscale.

As a preliminary analysis, we performed a MANOVA to assess whether participants’ sex and age affected gross motor skills. Sex and age were used as independent variables to investigate their combined and interactive effects on gross motor skills, which served as the dependent variable. MANOVA is designed to assess differences across multiple dependent variables simultaneously, offering valuable exploratory insights for subsequent network analysis, as this approach models the variables together. The multivariate test indicated an interaction of sex and chronological age (Hotelling’s trace = 1.54, *F*_(26,33946)_ = 1007.16, *p* < 0.001), which was observed in all individual skills of the TGMD-3: Run (*F*_(2,16986)_ = 2480.17, *p* < 0.001); Gallop (*F*_(2,16986)_ = 2702.72, *p* < 0.001); Hop (*F*_(2,16986)_ = 3201.57, *p* < 0.001); Skip (*F*_(2,16986)_ = 2606.86, *p* < 0.001); Horizontal jump (*F*_(2,16986)_ = 2051.86, *p* < 0.001); Slide (*F*_(2,16986)_ = 2608.49, *p* < 0.001); Two-hand strike of a stationary ball (*F*_(2,16986)_ = 2720.05, *p* < 0.001); Forehand strike of self-bounced (*F*_(2,16986)_ = 4037.14, *p* < 0.001); One-hand dribble stationary (*F*_(2,16986)_ = 5348.29, *p* < 0.001); Two-hand catch (*F*_(2,16986)_ = 3814.53, *p* < 0.001); Kick a stationary ball (*F*_(2,16986)_ = 3819.99, *p* < 0.001); Overhand throw (*F*_(2,16986)_ = 1903.55, *p* < 0.001); and Underhand throw (*F*_(2,16986)_ = 2670.44, *p* < 0.001). Accordingly, our subsequent analyses were performed considering age (stratified as 3–5, 6–8 and 9–11 years old) and sex. TGMD-3 scores of the sample, split by age and sex, are depicted in Fig. 1. Age was stratified into three groups to minimize variability within each group. The selected age ranges were chosen to effectively capture developmental differences, facilitating statistically significant comparisons. The stratification of ages 3–5, 6–8, and 9–11 years is based on well-established psychological and physical developmental milestones. Psychologically, these divisions align with Erikson’s psychosocial development theory, where children progress from initiative vs. guilt (ages 3–5), industry vs. inferiority (ages 6–8), and early identity formation (ages 9–11), shaping autonomy, academic self-efficacy, and peer interactions^33,34^. Physically, motor skill development follows a structured trajectory: children aged 3–5 refine fundamental movement skills such as running and jumping; those aged 6–8 enhance coordination, agility, and sports-related activities; while those aged 9–11 develop greater strength, endurance, and fine motor proficiency due to early pubertal changes^35^.

### Network construction

Network analysis provides an effective tool for grasping intricate and abstract structures like relationships and interactions, offering an intuitive way to understand them^36,37^. In a network, variables are referred to as “nodes”, connected by “edges” that can be weighted, with a higher weight indicating a stronger connection to a given node than an edge with a lower weight. Additionally, in undirected networks, this relationship is bi-directional. The nodes in the network for the present study comprise the skills measured using the TGMD-3, and their relationships are demonstrated by weighted and undirected edges that mirror the regularised partial correlations between them. These partial correlations are derived through the estimation of a Gaussian Graphical Model^38^ using a variant of the least absolute shrinkage and selection operator (LASSO) called graphical LASSO^39^. This technique reduces the occurrence of false positive connections by eliminating edges with values that are nearly zero^40,41^. The optimal model fitting is determined by selecting a tuning parameter through the minimization of the Extended Bayesian Criterion (EBIC), which is then controlled by a parameter γ^40^. This approach helps to identify the best-fitting model and ensures that the model is neither overfit nor underfit and the EBIC index is used to measure the trade-off between model fit and model complexity^42^. In our study, γ was set at 0.25 in accordance to the guidance provided by Epskamp^43^. Unlike direct calculations of partial correlations, graphical LASSO is useful for improving network sparsity^39^, emphasizing the most relevant and reliable relationships^44^.

### Network statistics

We computed centrality and bridge centrality statistics for each network, evaluating Strength centrality, Closeness centrality, and Betweenness centrality, along with their corresponding bridge centrality statistics^45,46^. Strength centrality measures a node’s communication ability based on its number of neighbours and the strength of its connections. Closeness centrality indicates the shortest path between a node and all other reachable nodes in the network, reflecting the node’s proximity to others. Betweenness centrality measures a node’s importance as a bridge between other nodes in the network. Bridge centrality statistics consider network communities, which represent subsets of highly interconnected nodes^47^ or a theoretically based group of nodes^45^. In our analyses, nodes were divided into two communities: Locomotor Skills and Ball Skills, as per the design of the TGMD-3^30^. Bridge centrality statistics encompass Strength centrality, Closeness centrality, and Betweenness centrality measures, but they specifically focus on a node’s relationships with communities outside its own. Bridge Strength assesses a node’s overall and direct connectedness to nodes in different communities. Bridge Betweenness measures how frequently a node falls on the shortest path between nodes belonging to separate communities. Bridge Closeness calculates the average distance between a given node and all other nodes outside its own community^45^. Centrality measures have been calculated as specified by Opsahl et al.^48^, considering the absolute values of the edges. The shortest path has been determined using Dijkstra’s algorithm^49^.

We estimated 95% Confidence Intervals (CIs) for each statistic by conducting 1000 non-parametric bootstraps for each network and calculating bootstrapped CIs for each statistic^38^. These CIs were used to evaluate the accuracy of the results, network statistics where the CIs do not span zero were considered accurate and reproducible. Additionally, we utilised the CIs to evaluate the differences between the groups in terms of network statistics, identifying significant differences where the CIs did not overlap. We used this approach to compare age groups and sex. Accordingly, in the presentation of the results, we report the average bootstrap values and the respective CIs. The stability of the networks’ indices was assessed via case-dropping subset bootstrap and the correlation stability coefficient (CS(cor = 0.70)), which indicates the highest proportion of cases that can be randomly dropped while maintaining a correlation > 0.70 with the original network statistics with 95% confidence. Values of the correlation stability coefficient > 0.25 were considered as acceptable^38^.

To the best of our knowledge, this study represents the first attempt to use this approach for comprehending the relationships among gross motor skills; therefore, we regarded all analyses as exploratory. Statistical analyses were performed with R^50^, using the *qgraph*^51^ and *bootnet*^52^ packages. Regularized partial correlations for each network have been provided as supplementary material (Table S1 to Table S10). Additionally, partial correlations between variables, calculated without regularisation, have been provided for comparison (Table S12 to Table S21).

## Results

### All-sample network

**Fig. 2:**
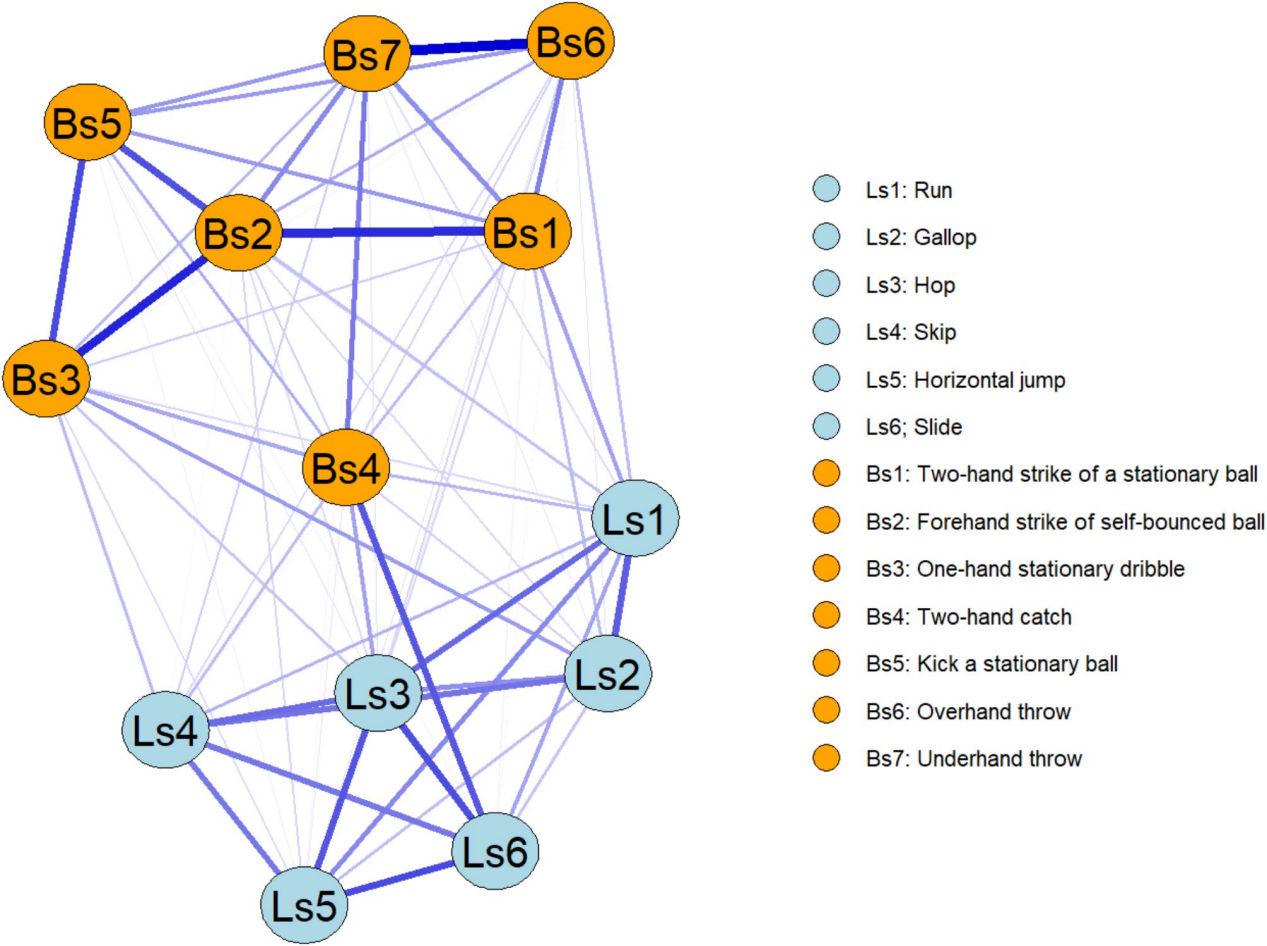
Network of the whole sample. Each node represents a measure of TGDM-3, the edges are represented by blue (positive correlation) or red dashed (negative correlation) lines, the connection weight is represented by the thickness of the line. Nodes that are more strongly connected are positioned closer together, while nodes with weaker connections are placed further apart. Network with fixed node positions is provided as supplementary material (Figure S1).

**Fig. 3:**
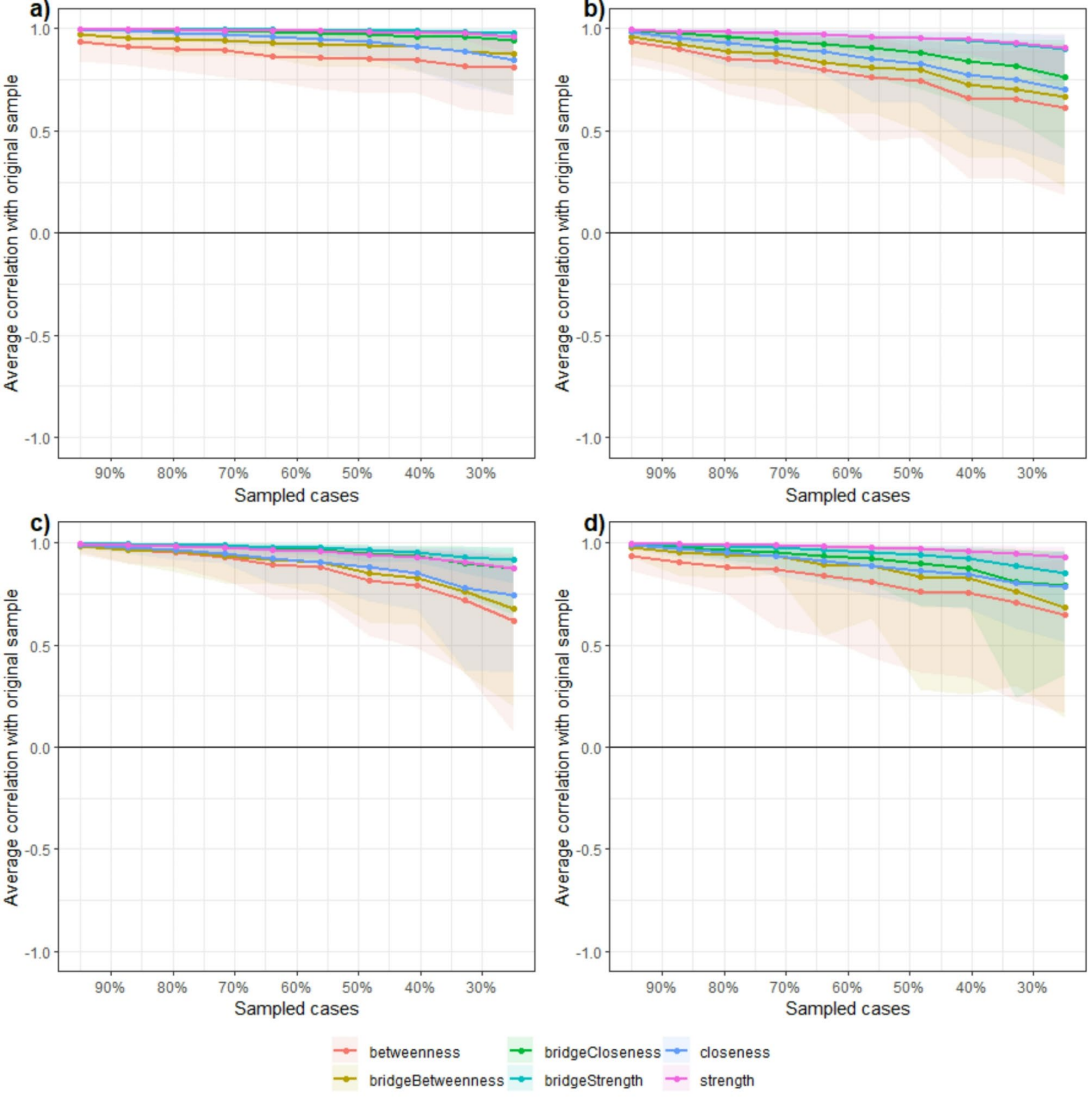
Results from case-drop bootstrap for; (**a**) All-sample network; (**b**) 3–5 years old children network; (**c**) 6–8 years old children network; (**d**) 9–11 years old children network. Shaded areas denote the range between the 2.5th and the 97.5th quantile.

The all-sample network (N = 16,989) is depicted in Fig. 2. Results indicated a good network stability (Fig. 3a) and every network statistic showed a correlation stability coefficient > 0.25.

**Fig. 4:**
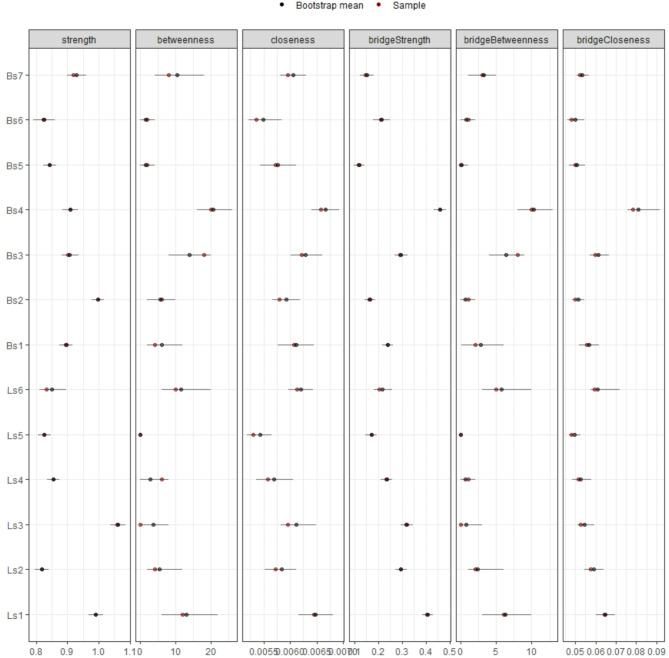
Centrality and bridge centrality statistics of the all-sample network. Coloured dots indicate original sample (red) and bootstrapped (blue) mean values, lines denote the range of the 95% CIs.

Figure 4 illustrates the centrality and bridge centrality statistics of the all-sample network. All nodes displayed non-zero values within their CIs for strength and closeness centrality, as well as for bridge strength and bridge closeness (Fig. 4). However, this was not the case for betweenness centrality and bridge betweenness, and some nodes had zero in their CIs; specifically, hopping, skipping, horizontal jumping, kicking, and overhand throwing for betweenness centrality; and hopping, skipping, horizontal jumping, forehand striking, kicking stationary ball, and overhand throwing for bridge betweenness. This suggests poor accuracy of these values and calls for caution in their interpretation. Detailed mean values of the network statistics and their corresponding CIs for both the all-sample network and the age groups networks are reported in detail in Table 1.

**Table 1:** Mean values of networks statistics, along with their confidence intervals [95% CIs], are reported for both the all-sample network and the age groups networks. 95% CIs that do not include zero are in bold.

Groups	Ls1_Run	Ls2_Gallop	Ls3_Hop	Ls4_Skip	Ls5_Horizontal Jump	Ls6_Slide	Bs1_Two-hand strike	Bs2_Forehand strike	Bs3_One-hand stationary	Bs4_Two-hand catch	Bs5_Kick stationary ball	Bs6_Overhand throw	Bs7_Underhand throw
*Strength centrality*													
All-sample	**0.99** **[0.96–1.01]**	**0.81** **[0.79–0.84]**	**1.06** **[1.03–1.08]**	**0.85** **[0.83–0.87]**	**0.82** **[0.80–0.84]**	**0.85** **[0.80–0.89]**	**0.89** **[0.87–0.91]**	**0.99** **[0.97–1.01]**	**0.90** **[0.88–0.93]**	**0.90** **[0.88–0.93]**	**0.84** **[0.82–0.86]**	**0.82** **[0.79–0.86]**	**0.92** **[0.89–0.96]**
3–5 y.o	1.01*^+^ **[0.93–1.12]**	0.81*^+^ **[0.73–0.90]**	1.05*^+^ **[0.99–1.10]**	0.97*^+^ **[0.86–1.11]**	**0.74** **[0.69–0.79]**	0.90*^+^ **[0.84–1.00]**	1.01*^+^ **[0.93–1.11]**	**0.88** **[0.82–0.96]**	**0.74** **[0.68–0.79]**	0.76*^+^ **[0.70–0.86]**	**0.85** **[0.75–0.96]**	**0.84** **[0.79–0.89]**	0.92*^+^ **[0.87–1.00]**
6–8 y.o	0.78* **[0.74–0.83]**	0.64*° **[0.60–0.70]**	0.84* **[0.79–0.89]**	0.68* **[0.62–0.76]**	**0.79** **[0.75–0.85]**	0.71* **[0.62–0.81]**	0.72* **[0.67–0.77]**	**0.88** **[0.83–0.95]**	**0.78** **[0.72–0.84]**	0.63*° **[0.58–0.68]**	**0.75** **[0.70–0.83]**	**0.76** **[0.70–0.82]**	0.80* **[0.74–0.86]**
9–11 y.o	**0.75**^**+**^ **[0.69–0.82]**	**0.50°**^**+**^ **[0.43–0.58]**	**0.78**^**+**^ **[0.71–0.85]**	**0.59**^**+**^ **[0.52–0.67]**	**0.81** **[0.75–0.89]**	**0.65**^**+**^ **[0.56–0.77]**	**0.68**^**+**^ **[0.62–0.76]**	**0.85** **[0.78–0.92]**	**0.66** **[0.58–0.74]**	**0.44°**^**+**^ **[0.37–0.51]**	**0.70** **[0.63–0.77]**	**0.88** **[0.82–0.94]**	**0.77**^**+**^ **[0.71–0.84]**
*Closeness centrality*													
All-sample	**0.0064** **[0.0061–0.0068]**	**0.0058** **[0.0055–0.0061]**	**0.0061** **[0.0058–0.0064]**	**0.0056** **[0.0053–0.0060]**	**0.0054** **[0.0051–0.0056]**	**0.0062** **[0.0059–0.0064]**	**0.0061** **[0.0057–0.0064]**	**0.0059** **[0.0056–0.0061]**	**0.0062** **[0.0060–0.0066]**	**0.0066** **[0.0064–0.0069]**	**0.0057** **[0.0054–0.0061]**	**0.0054** **[0.0052–0.0058]**	**0.0060** **[0.0058–0.0063]**
3–5 y.o	**0.0068** **[0.0062–0.0076]**	**0.0061**^**+**^ **[0.0054–0.0068]**	0.0069*^+^ **[0.0063–0.0075]**	0.0067*^+^ **[0.0061–0.0074]**	**0.0059** **[0.0054–0.0065]**	0.0063*^+^ **[0.0058–0.0069]**	0.0070*^+^ **[0.0064–0.0076]**	0.0064*^+^ **[0.0059–0.0069]**	**0.0064**^**+**^ **[0.0058–0.0069]**	0.0064*^+^ **[0.0059–0.0069]**	**0.0059** **[0.0054–0.0065]**	0.0061* **[0.0055–0.0068]**	0.0065*^+^ **[0.0059–0.0072]**
6–8 y.o	**0.0060** **[0.0056–0.0063]**	**0.0052** **[0.0048–0.0056]**	0.0054* **[0.0051–0.0058]**	0.0046* **[0.0042–0.0050]**	**0.0051** **[0.0047–0.0055]**	0.0050* **[0.0046–0.0054]**	0.0047* **[0.0043–0.0052]**	0.0053* **[0.0049–0.0057]**	**0.0053** **[0.0049–0.0058]**	0.0052*° **[0.0047–0.0056]**	**0.0053** **[0.0048–0.0057]**	0.0050* **[0.0045–0.0054]**	0.0052* **[0.0047–0.0056]**
9–11 y.o	**0.0057** **[0.0052–0.0063]**	**0.0045**^**+**^ **[0.0040–0.0051]**	**0.0053**^**+**^ **[0.0047–0.0058]**	**0.0043**^**+**^ **[0.0039–0.0048]**	**0.0051** **[0.0047–0.0056]**	**0.0047**^**+**^ **[0.0042–0.0054]**	**0.0046**^**+**^ **[0.0042–0.0052]**	**0.0050**^**+**^ **[0.0045–0.0056]**	**0.0049**^**+**^ **[0.0043–0.0056]**	**0.0040°**^**+**^ **[0.0035–0.0046]**	**0.0051** **[0.0046–0.0056]**	**0.0049** **[0.0042–0.0055]**	**0.0047**^**+**^ **[0.0042–0.0053]**
*Betweenness centrality*													
All-sample	**13.08 [6–22]**	**5.33 [2–12]**	3.56 [0–8]	2.77 [0–8]	0.02 [0–0]	**11.45 [6–20]**	**6.07 [2–12]**	**5.54 [2–10]**	**13.84 [8–20]**	**20.58 [16–26]**	1.57 [0–4]	1.40 [0–4]	**10.5 [4–18]**
3–5 y.o	**12.28 [2–26]**	1.99 [0–8]	**16.21 [6–26]**	**9.30 [2–18]**	0.79 [0–4]^+^	4.02 [0–12]	**14.27 [6–26]**	**6.81 [2–16]**	**11.73 [2–20]**	**9.38 [4–16]**	3.92 [0–10]	2.18 [0–10]	**10.31 [2–22]**
6–8 y.o	**21.44 [10–32]**	8.40 [0–16]	**10.90 [2–18]**	1.44 [0–6]	2.62 [0–12]	**9.85 [4–16]**	0.21 [0–4]	**8.91 [4–18]**	**11.56 [4–20]**	**13.06 [4–20]**	3.35 [0–12]	4.01 [0–10]	**11.77 [4–20]**
9–11 y.o	**23.05 [8–40]**	2.68 [0–10]	8.15 [0–20]	2.95 [0–10]	**16.17 [6–28]**^**+**^	**7.84 [2–18]**	1.71 [0–10]	**10.36 [2–24]**	4.79 [0–16]	1.94 [0–8]	6.64 [0–18]	**13.30 [4–24]**	7.73 [0–18]
*Bridge strength*													
All-sample	**0.40** **[0.38–0.42]**	**0.29** **[0.27–0.32]**	**0.31** **[0.29–0.34]**	**0.23** **[0.21–0.26]**	**0.17** **[0.14–0.19]**	**0.21** **[0.18–0.25]**	**0.23** **[0.21–0.26]**	**0.16** **[0.14–0.18]**	**0.29** **[0.26–0.32]**	**0.45** **[0.43–0.48]**	**0.12** **[0.09–0.14]**	**0.21** **[0.17–0.24]**	**0.15** **[0.12–0.18]**
3–5 y.o	**0.31** **[0.24–0.41]**	**0.17** **[0.09–0.25]**	0.36*^+^ **[0.31–0.42]**	**0.27** **[0.19–0.39]**	**0.15** **[0.10–0.21]**	**0.19** **[0.14–0.27]**	0.35*^+^ **[0.28–0.44]**	**0.11** **[0.05–0.18]**	**0.28** **[0.23–0.33]**	**0.29** **[0.23–0.37]**	**0.09** **[0.01–0.19]**	**0.19** **[0.14–0.24]**	**0.14** **[0.10–0.21]**
6–8 y.o	**0.36** **[0.32–0.40]**	**0.22** **[0.18–0.26]**	0.17* **[0.13–0.22]**	**0.18** **[0.13–0.25]**	**0.16** **[0.11–0.20]**	**0.25** **[0.19–0.32]**	0.16* **[0.11–0.21]**	**0.13** **[0.09–0.18]**	**0.28** **[0.23–0.033]**	**0.33** **[0.29–0.38]**	**0.11** **[0.06–0.17]**	**0.21** **[0.16–0.26]**	**0.12** **[0.07–0.17]**
9–11 y.o	**0.36** **[0.31–0.42]**	**0.21** **[0.16–0.26]**	**0.19**^**+**^ **[0.15–0.25]**	**0.13** **[0.08–0.19]**	**0.14** **[0.09–0.20]**	**0.18** **[0.11–0.27]**	**0.10**^**+**^ **[0.05–0.16]**	**0.16** **[0.11–0.22]**	**0.20** **[0.13–0.27]**	**0.26** **[0.20–0.32]**	**0.21** **[0.16–0.26]**	**0.19** **[0.14–0.24]**	**0.10** **[0.06–0.15]**
*Bridge closeness*													
All-sample	**0.064** **[0.060–0.059]**	**0.058** **[0.054–0.064]**	**0.054** **[0.051–0.059]**	**0.052** **[0.048–0.057]**	**0.049** **[0.046–0.052]**	**0.060** **[0.057–0.071]**	**0.056** **[0.051–0.061]**	**0.051** **[0.048–0.054]**	**0.061** **[0.057–0.066]**	**0.080** **[0.075–0.091]**	**0.050** **[0.046–0.054]**	**0.049** **[0.046–0** **.054]**	**0.053** **[0.050–0.056]**
3–5 y.o	**0.065** **[0.057–0.074]**	**0.057** **[0.050–0.065]**	0.067*^+^ **[0.059–0.076]**	0.064*^+^ **[0.0056–0.072]**	**0.056** **[0.050–0.064]**	0.058*^+^ **[0.052–0065]**	0.068*^+^ **[0.059–0.078]**	0.059* **[0.053–0.064]**	0.069*^+^ **[0.060–0.079]**	**0.073** **[0.063–0.082]**	**0.050** **[0.045–0.054]**	**0.056** **[0.049–0.066]**	0.058*^+^ **[0.051–0.068]**
6–8 y.o	**0.062** **[0.056–0.067]**	**0.051** **[0.046–0.056]**	0.048* **[0.045–0.052]**	0.042* **[0.038–0.047]**	**0.046** **[0.042–0.051]**	0.047* **[0.042–0.051]**	0.042* **[0.037–0.048]**	0.046* **[0.042–0.051]**	0.053* **[0.047–0.059]**	**0.067** **[0.061–0.073]**	**0.049** **[0.043–0.055]**	**0.047** **[0.040–0.053]**	0.045* **[0.040–0050]**
9–11 y.o	**0.059** **[0.0052–0.067]**	**0.047** **[0.040–0.054]**	**0.049**^**+**^ **[0.043–0.056]**	**0.039**^**+**^ **[0.035–0.045]**	**0.046** **[0.041–0.051]**	**0.041**^**+**^ **[0.037–0.045]**	**0.041**^**+**^ **[0.036–0.049]**	**0.045** **[0.038–0.054]**	**0.044**^**+**^ **[0.038–0.054]**	**0.057** **[0.048–0.066]**	**0.053** **[0.045–0.061]**	**0.044** **[0.038–0.051]**	**0.042**^**+**^ **[0.035–0.049]**
*Bridge betweenness*													
All-sample	**6.28 [3–10]**	**2.38 [1–6]**	0.72 [0–3]	0.63 [0–2]	0.01 [0–0]	**5.72 [3–10]**	**2.81 [0.02–6]**	0.58 [0–2]	**6.39 [4–9]**	**10.29 [8–13]**	0.02 [0–1]	0.69 [0–2]	**3.17 [1–5]**
3–5 y.o	**5.45 [1–12]**	0.79 [0–4]	**7.59 [2–13]**	3.90 [0–8]	0.29 [0–2]^+^	1.39 [0–5]	**6.23 [2–11]**	0.95 [0–4]	**5.90 [1–10]**	**4.81 [2–8]**	0.08 [0–1]	0.90 [0–4]	2.35 [0–7]
6–8 y.o	**10.79 [6–16]**	4.04 [0–8]	3.15 [0–7]	0.58 [0–2]	0.92 [0–4]	**4.88 [2–8]**	0.08 [0–1]	1.78 [0–6]	**5.24 [2–9]**	**6.92 [3–10]**	1.09 [0–5]	1.90 [0–5]	2.47 [0–5]
9–11 y.o	**23.23 [5–21]**	1.55 [0–5]	3.09 [0–9]	0.66 [0–4]	**7.19 [3–12]**^**+**^	2.33 [0–5]	0.56 [0–5]	3.22 [0–10]	0.62 [0–4]	1.17 [0–4]	4.45 [0–10]	3.64 [0–8]	1.19 [0–5]

*Indicates a difference between the 3–5 years old (y.o.) group network and the 6–8 years old group network, ° indicates a difference between the 6–8 years old group network and the 9–11 years old group network, ^+^ indicates a difference between the 3–5 years old group network and the 9–11 years old group network.

Based on our findings, hopping displayed higher strength centrality than other nodes, followed by forehand striking and running. This suggests that these nodes have stronger connections with their neighbouring nodes (Fig. 4). Additionally, two-hand catching, running, and one-hand stationary dribbling exhibited higher values of closeness centrality than other nodes, indicating they were closer to all other nodes in the network; as evidenced by their centrality in the network (Fig. 2). This suggests that these motor skills are more closely related to all other nodes. Furthermore, in terms of betweenness centrality, two-hand catching had higher values than other nodes, followed by running and one-hand stationary dribbling (Fig. 4), representing important “bridges” between other nodes in the network. These results suggest the importance of these nodes in the all-sample network, as they exhibit stronger, closer relationships with other nodes and play crucial linking roles.

Regarding bridge centrality statistics, two-hand catching exhibited higher values of bridge strength than all other nodes, followed by running, and hopping (Fig. 4). This indicates that these nodes have the strongest direct connections to the other community (i.e., two-hand catching had the greatest relationship on locomotor skills, and running and hopping had the greatest relationship on ball skills). Nodes with non-zero values of bridge betweenness are highlighted in Table 1. Two-hand catching exhibited the highest values of bridge betweenness, followed by one-hand stationary dribbling and running. This highlights the role of two-hand catching in connecting nodes from two different communities, serving as the most important bridge between them. Running, even if with a lesser value, serves as the counterpart among Locomotor skills.

### Network comparisons by age

**Fig. 5:**
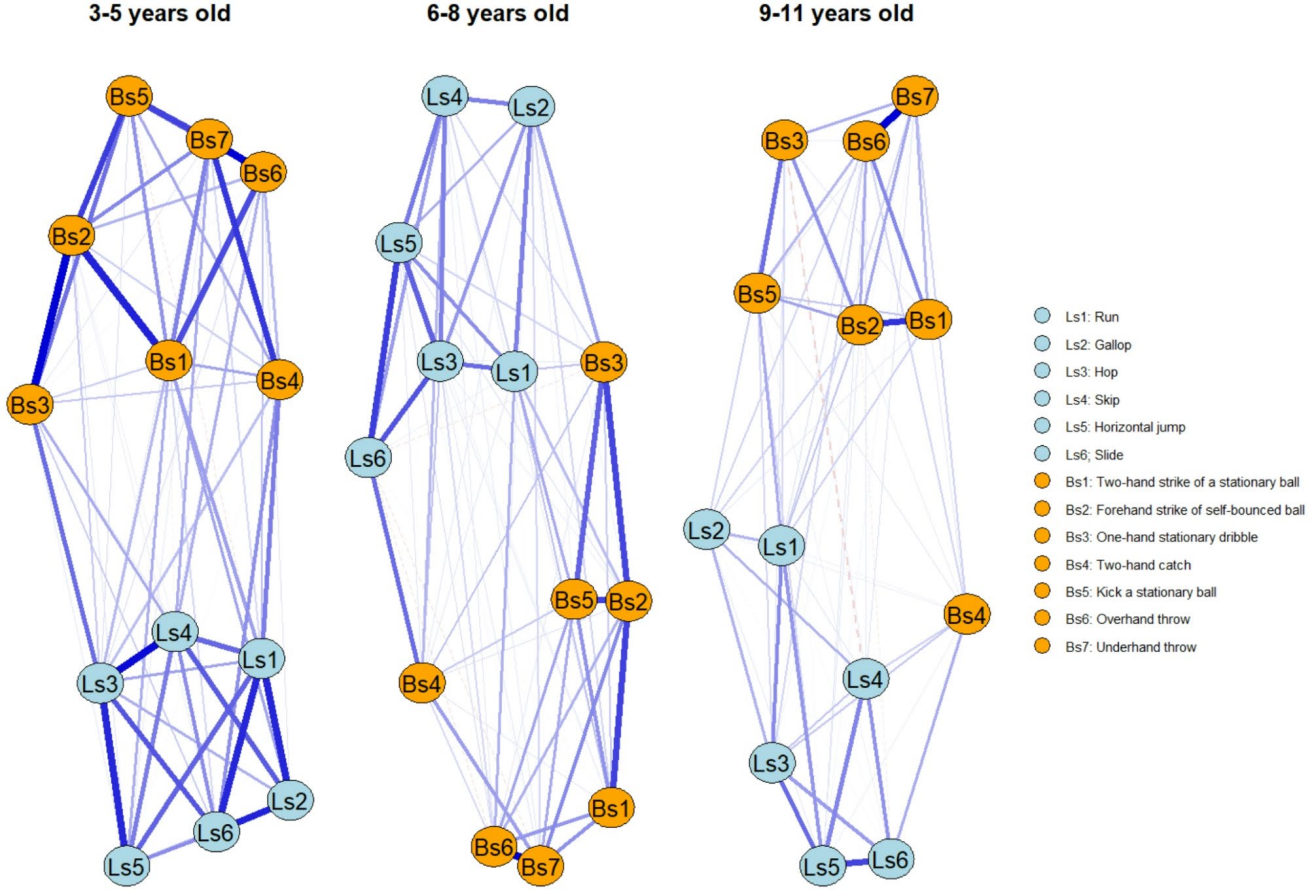
Network of the three age groups. Each node represents a measure of TGDM-3, the edges are represented by blue (positive correlation) or red dashed (negative correlation) lines, the connection weight is represented by the thickness of the line. Nodes that are more strongly connected are positioned closer together, while nodes with weaker connections are placed further apart. Networks with fixed node positions are provided as supplementary material (Figure S1).

Figure 5 presents the networks stratified by age (N_3-5y_ = 3525, N_6-8y_ = 7882, N_9-11y_ = 5582). These networks demonstrated good stability (Fig. 3). Additionally, all of the statistics displayed a CS(cor = 0.70) > 0.25, with the 3–5 and 9–11 age groups showing the lowest on betweenness centrality (CS(cor = 0.70) = 0.28, for both). This indicates that the data demonstrates high consistency across all statistics, signified by a stability coefficient greater than 0.25. This consistency confirms the results are stable and reliable. Detailed values of networks statistics and comparisons between age group are reported in Table 1.

**Fig. 6:**
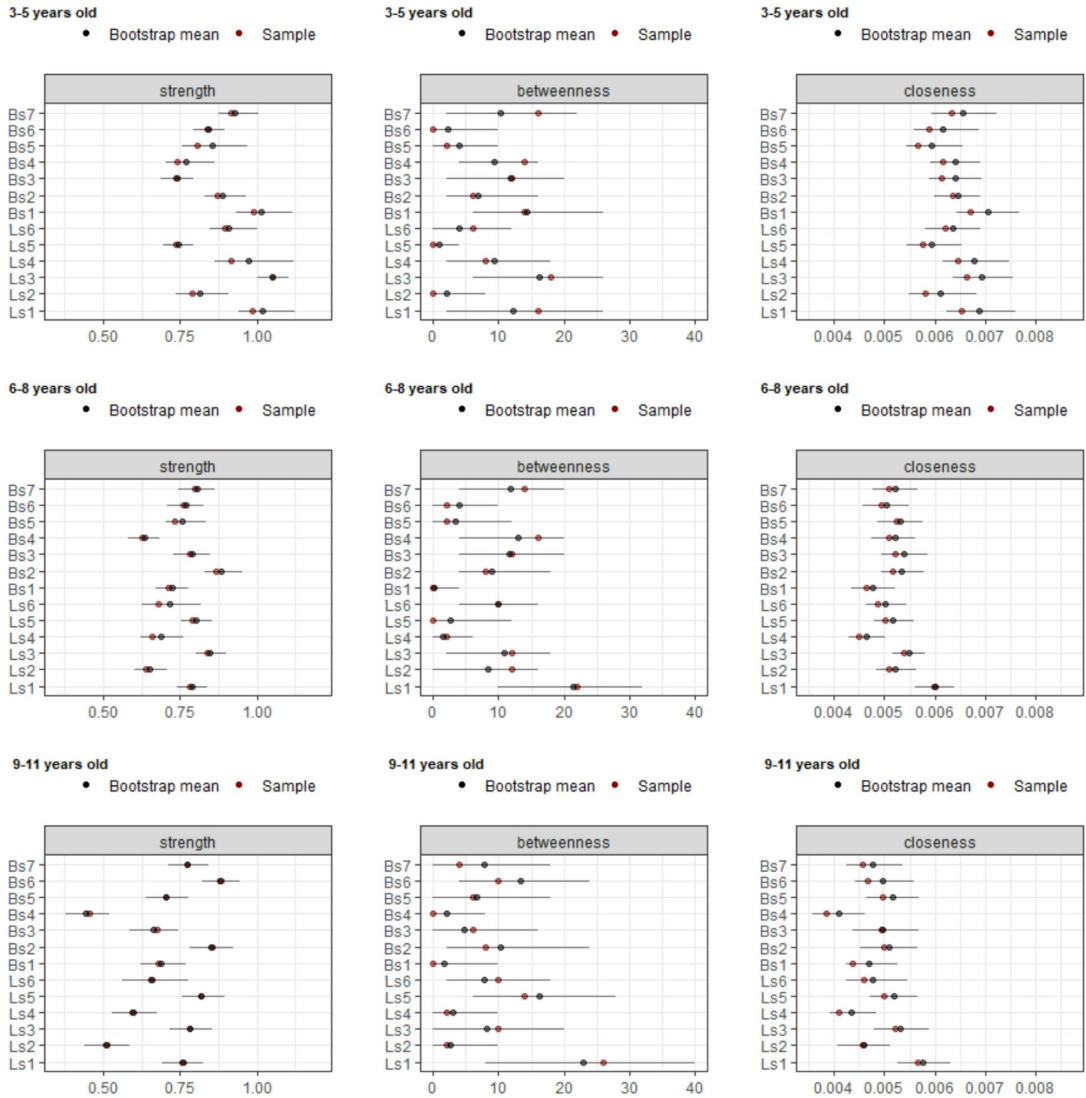
Centrality statistics of the age groups network. Coloured dots indicate original sample (red) and bootstrapped (blue) mean values, lines denote the range of the 95% CIs.

**Fig. 7:**
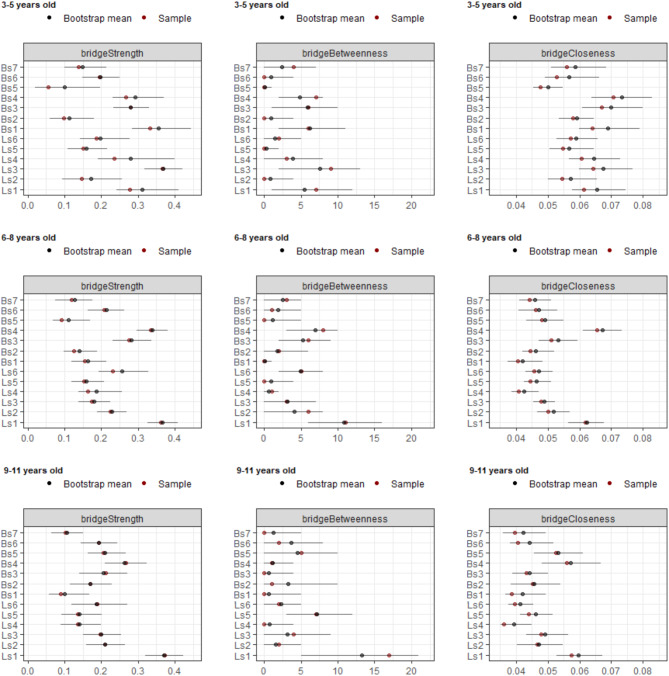
Bridge centrality statistics of the age groups network. Coloured dots indicate original sample (red) and bootstrapped (blue) mean values, lines denote the range of the 95% CIs.

All nodes showed non-zero values within their CIs on strength and closeness centrality, as well as for bridge strength and bridge closeness, while for betweenness centrality and bridge betweenness this was affected by age (Figs. 6 and 7). Regarding betweenness, findings indicated that running appeared to be particularly important, as it did not have a zero value among its CIs in all age groups. Similarly, the forehand striking node was also found to be important across all age groups, with no zero values in its CIs. Regarding bridge betweenness, only running did not contain zero among its CIs in all age group networks (Table 1). This suggests that for all age groups, running seems particularly important in bridging nodes in the network and between communities.

Group comparisons revealed meaningful insights (Table 1). Regarding centrality statistics, results indicated a trend where strength and closeness statistics decreased with age, with significant differences between age groups. Specifically, the 3–5 year old network showed higher (i.e. non-overlapping CIs) centrality strength values compared to the 6–8 year old network on Locomotor skills such as running, galloping, hopping, skipping, sliding, and on Ball skills such as two-hand striking of a stationary ball, two-hand catching, and underhand throwing. Differences were also found between the 6–8 and 9–11 year old group networks, where strength was lower on galloping and two-hand catching in the older age group. Results showed a reduction of closeness centrality between 3–5 and 6–8 year old networks on Locomotor skills as hopping, skipping, and sliding and on Ball skills such as two-hand striking of a stationary ball, forehand striking, two-hand catching, overhand throwing and underhand throwing. There were no significant differences in betweenness centrality values between different age groups. However, overall, these results suggest that the nodes tend to get further apart during development (i.e., the relationships between gross motor skills are strongest in young children and get weaker as children age). The reliability of the results is confirmed by the correlation stability coefficient, which demonstrated robustness under varying weight distributions caused by subsampling.

A similar trend was found regarding bridge centrality statistics. There were differences between 3–5 and 6–8 year old networks on bridge strength for hopping and two-hand striking, with the younger group having higher values. Whilst there were no differences between the 6–8 and 9–11 year old groups, it is interesting to report that in the 9–11 year old network, running has the highest significant bridge strength compared to all other nodes (Fig. 7). There were differences between the 3–5 year old group and the 6–8 year old networks regarding bridge closeness, where the older group showed lower values on Locomotor skills such as hopping, skipping, sliding, and on Ball skills such as two hand striking, forehand striking, one-hand stationary dribbling, and underhand throwing; suggesting an increase of the average distance between nodes of different communities in older age groups.

### Network comparisons by sex

Results concerning sex are presented in the supplementary material. The findings indicated that for betweenness statistics of 9–11 year old boys and betweenness and bridge betweenness of all three age groups of girls, the CS (cor = 0.70) values were less than 0.25. This suggests that caution should be exercised when interpreting results related to these statistics. Networks are depicted in Supplementary Figure S2 and Figure S3. The results for male and female, when analysed separately by age groups, revealed the same trend observed in the previous networks; whereby there was a decrease in network strength accompanied by a reduction in node closeness in the older age groups (Supplementary Figures S4 to S7, and Table S11). A Chi-square test also showed that there is no statistically significant difference in the distribution of boys and girls across the age groups (Chi-square(2) = 5.60, *p* = 0.061), indicating the sample distribution of boys and girls is balanced across the age groups.

## Discussion

This study examines the relationship between gross motor skills through network analysis. The findings shed light on a robust interconnectedness among gross motor skills, where locomotor skills and ball skills exhibit particularly strong associations with other skills within their respective categories. Notably, two-hand catching (a Ball Skill) and running (a Locomotor Skill) emerge as pivotal elements. Furthermore, as individuals age, these interconnections between skills tend to weaken, leading to a greater degree of independence between them. The results also demonstrate that similar patterns can be observed in the relationships in gross motor skills between boys and girls.

The network analysis indicated important characteristics of the relations between gross motor skills. From the graphical representations of the overall sample network, it can be observed that all the nodes in the network are highly connected (Fig. 2), confirming that all 13 gross motor skills examined in the TGMD-3 are strongly related. It can also be observed that Locomotor skills and Ball skills tends to cluster by themselves, confirming these as two different sets of gross motor skills^30,31^.

According to the network analysis results, *two-hand catch* (a Ball Skill) and *running* (a Locomotor Skill) seem particularly important. It can be observed that *two hand catch* and *running* (Fig. 2) tend to be in the middle of the network, with their importance supported by the network statistics. We can therefore assume that the other gross motor skills rely, to some extent, on the development of these two main skills. Indeed, *two-hand catch* demonstrates greater values than other nodes in terms of closeness and bridge closeness, as well as betweenness and bridge betweenness. It also has higher values for bridge strength compared to other nodes. This indicates that it is highly connected with all the other skills in its own community (i.e., Ball skills) and in the other community (i.e., Locomotor skills). Additionally, the high values in centrality betweenness and bridge betweenness suggest that *two-hand catch* serves as an important “bridge” between skills within its community and with others. Catching a ball is a multifaceted motor skill that necessitates precise adjustment of hand movement force and timing in response to the ball’s direction, speed, weight, and size^53^, and to complete a successful catch the motions preparation and initiation must be seamlessly integrated within a controlled body posture^54^. Moreover, in terms of biomechanics, arm coordination is associated with locomotor performance, as arm swing is linked to performance in both the maximum jump distance and the horizontal aspects of the ground reaction force^55^. Thus, the gross motor skill *two-hand catch* is an important fundamental for the development of other gross motor skills, as evidenced by the network statistics in the present study.

Similarly, *running* consistently exhibited higher values compared to most other nodes in all considered statistics, indicating that it is one of the most influential skills in the network. Running is a transition from walking and many of the motion parameters between these tasks are highly related^56^. The developmental transition from walking to running is related to many mechanisms such as neural control, sensory control, and strength and postural control capacities^56,57^. Furthermore, *running* includes both leg and arm actions, synchronised with the leg pattern^8^. Evidence suggests that children typically become proficient runners during pre-school, before mastering other gross motor skills^1^. Thus, given its importance and relationship with the early stages of motor and brain development^58^, the processes involved in running skills are highly associated with other gross motor skills.

Results indicated important differences in the relationships between gross motor skills during development and ageing. Overall, the network statistics indicated a trend where strength and bridge strength were lower in the older age groups, alongside lower values for closeness and bridge closeness. These findings suggest that younger children have stronger and closer connections between their gross motor skills, which decrease with ageing and development. This suggests that during development each gross motor skill becomes more independent as the associations grow weaker, and the nodes become more distant. Indeed, as children become more proficient, specific movements are acquired and specific motor programs are developed to pursue specific goal-oriented actions^59–61^, likely occurring with greater specialisation to specific sports and activities. The results of the network analyses also indicated similar relationships between gross motor skills in boys and girls. This suggests that, despite potential differences in performance^25^, the relationships and interactions of gross motor skills on each other is similar between boys and girls. Thus, it is likely that both boys and girls aged 3–11 could benefit from educational practices focused on gross motor skills in a similar manner. It is noteworthy that, to some extent, this supports the hypothesis that differences between boys and girls during childhood could be due to the kind of activities they typically participate in^25^.

To the best of our knowledge, this is the first study that analysed the dynamics among gross motor skills during the development of 3 to 11 year old children using network analysis. One particular strength of study is related to the sample size of the participants (~ 17,000 children), indeed, the networks obtained showed high stability, indicating high replicability of the results^40^. However, the present study is also not without limitation. For example we analysed differences between age groups in a cross-sectional design, thus it is not possible to exclude the effect of confounding variables (although this is unlikely in such a large sample). In future work, a longitudinal design should be considered to track the development of gross motor skills with ageing, and to track the changing nature of the relationships between gross motor skills. To further extend the present study (conducted in children aged 3–11 years old), future studies should also consider older age groups, and how gross motor skills track into adolescence and adulthood. In adolescence, it would be particularly interesting and pertinent to examine sex differences in gross motor skill networks due to the differing hormonal and developmental profiles of boys and girls during this time. While the correlation stability coefficient demonstrated robustness under varying weight distributions within each age group, it does not fully account for potential differences in weight distributions across groups. Therefore, caution should be exercised when interpreting between-group comparisons.

In conclusion, *two-hand catching* and *running* were identified as particularly important gross motor skills in our network analyses. For this reason, these two skills should be given special attention during the early stages of fundamental motor skill development, and emphasised in educational, clinical, and intervention contexts. The findings of the present study also demonstrate developmental changes in the relationship between gross motor skills; whereby the relationships between gross motor skills are strongest in the youngest age group (3–5 y) and get weaker as children get older. Finally, the present study demonstrates similar networks of gross motor skills in boys and girls aged 3–11 years old; suggesting that the relationships between gross motor skills are not different between boys and girls at this age. Overall, the findings of the present study provide important, novel, evidence of the relationships between gross motor skills during childhood, and how these change as children get older. These findings have important implications for the teaching of gross motor skills and ensuring optimal gross motor skill development in young people.

## Electronic supplementary material

Below is the link to the electronic supplementary material.

Supplementary Material 1

## Data Availability

Anonymised participant data will be made available on reasonable request to the corresponding author.

## References

[1] Hardy, L. L., King, L., Farrell, L., Macniven, R. & Howlett, S. Fundamental movement skills among Australian preschool children. *J. Sci. Med. Sport* **13**, 503–508 (2010). 19850520 10.1016/j.jsams.2009.05.010

[2] Monsma, H. G. W., Eva V. Assessment of Gross Motor Development. in *Psychoeducational Assessment of Preschool Children* (Routledge, 2007).

[3] Payne, V. G. & Isaacs, L. D. *Human Motor Development: A Lifespan Approach*. (Routledge, 2017).

[4] Woodfield, L. *Physical Development in the Early Years*. (Bloomsbury Publishing, 2004).

[5] Logan, S. W., Ross, S. M., Chee, K., Stodden, D. F. & Robinson, L. E. Fundamental motor skills: A systematic review of terminology. *J. Sports Sci.* **36**, 781–796 (2018). 28636423 10.1080/02640414.2017.1340660

[6] Cattuzzo, M. T. et al. Motor competence and health related physical fitness in youth: A systematic review. *J. Sci. Med. Sport* **19**, 123–129 (2016). 25554655 10.1016/j.jsams.2014.12.004

[7] Martins, C. et al. Motor competence and body mass index in the preschool years: A pooled cross-sectional analysis of 5545 children from eight countries. *Sports Med.*10.1007/s40279-023-01929-7 (2023). 37747664 10.1007/s40279-023-01929-7 PMC10939976

[8] Goodway, J. D., Ozmun, J. C. & Gallahue, D. L. *Understanding Motor Development: Infants, Children, Adolescents, Adults*. (Jones & Bartlett Learning, 2019).

[9] Mohammadi Orangi, B. *et al.* Emotional intelligence and motor competence in children, adolescents, and young adults. *Eur. J. Dev. Psychol.* **20**, 66–85 (2023).

[10] Ghassabian, A. et al. Gross motor milestones and subsequent development. *Pediatrics* **138**, e20154372 (2016). 27354457 10.1542/peds.2015-4372 PMC4925077

[11] Boat, R. et al. 16 weeks of physically active mathematics and English language lessons improves cognitive function and gross motor skills in children aged 8–9 years. *Int. J. Environ. Res. Public. Health* **19**, 16751 (2022). 36554632 10.3390/ijerph192416751 PMC9779825

[12] Magistro, D. et al. Two years of physically active mathematics lessons enhance cognitive function and gross motor skills in primary school children. *Psychol. Sports Exerc.* **63**, 102254 (2022).

[13] Piek, J. P., Dawson, L., Smith, L. M. & Gasson, N. The role of early fine and gross motor development on later motor and cognitive ability. *Hum. Mov. Sci.* **27**, 668–681 (2008). 18242747 10.1016/j.humov.2007.11.002

[14] Magistro, D., Bardaglio, G. & Rabaglietti, E. Gross Motor Skills and Academic Achievement in Typically Developing Children: The Mediating Effect of Adhd Related Behaviours. *Cogn. Creier Comportament Cognition Brain Behav.* **19** (2015).

[15] van der Fels, I. M. J. et al. The relationship between motor skills and cognitive skills in 4–16 year old typically developing children: A systematic review. *J. Sci. Med. Sport* **18**, 697–703 (2015). 25311901 10.1016/j.jsams.2014.09.007

[16] Metcalfe, J. & Clark, J. The mountain of motor development: A metaphor. *Motor Dev. Res. Rev.* **2**, 163–190 (2002).

[17] Sgrò, F., Quinto, A. M. V., Messana, L., Pignato, S. & Lipoma, M. Assessment of gross motor developmental level in Italian primary school children. *J. Phys. Educ. Sport* **17**, 1954–1959 (2017).

[18] Brian, A. et al. Motor competence levels and developmental delay in early childhood: A multicenter cross-sectional study conducted in the USA. *Sports Med.* **49**, 1609–1618 (2019). 31301035 10.1007/s40279-019-01150-5

[19] Wilson, P. H., Ruddock, S., Smits-Engelsman, B., Polatajko, H. & Blank, R. Understanding performance deficits in developmental coordination disorder: a meta-analysis of recent research. *Dev. Med. Child Neurol.* **55**, 217–228 (2013). 23106668 10.1111/j.1469-8749.2012.04436.x

[20] Adolph, K. E. & Robinson, S. R. Motor Development. in *Handbook of Child Psychology and Developmental Science* (ed. Lerner, R. M.) 1–45 (Wiley, 2015). 10.1002/9781118963418.childpsy204.

[21] Govatos, L. A. Relationships and age differences in growth measures and motor skills. *Child Dev.* **30**, 333–340 (1959). 13828627 10.1111/j.1467-8624.1959.tb04942.x

[22] Henry, F. M. & Nelson, G. A. Age differences and inter-relationships between skill and learning in gross motor performance of ten- and fifteen-year-old boys. *Res. Q. Am. Assoc. Health Phys. Educ. Recreat.* (1956).

[23] Barnett, L. M. et al. Correlates of gross motor competence in children and adolescents: A systematic review and meta-analysis. *Sports Med. Auckl. NZ* **46**, 1663–1688 (2016). 10.1007/s40279-016-0495-z PMC5055571 26894274

[24] Bala, G., Jaksic, D. & Katić, R. Trend of relations between morphological characteristics and motor abilities in preschool children. *Coll. Antropol.* **33**, 373–385 (2009). 19662753

[25] Bolger, L. E. et al. Global levels of fundamental motor skills in children: A systematic review. *J. Sports Sci.* **39**, 717–753 (2021). 33377417 10.1080/02640414.2020.1841405

[26] Behan, S., Belton, S., Peers, C., O’Connor, N. E. & Issartel, J. Moving Well-Being Well: Investigating the maturation of fundamental movement skill proficiency across sex in Irish children aged five to twelve. *J. Sports Sci.* **37**, 2604–2612 (2019). 31379260 10.1080/02640414.2019.1651144

[27] Bolger, L. E. et al. Age and sex differences in fundamental movement skills among a cohort of irish school children. *J. Mot. Learn. Dev.* **6**, 81–100 (2018).

[28] Kelly, L., O’Connor, S., Harrison, A. J. & Ní Chéilleachair, N. J. Does fundamental movement skill proficiency vary by sex, class group or weight status? Evidence from an Irish primary school setting. *J. Sports Sci.* **37**, 1055–1063 (2019). 10.1080/02640414.2018.1543833 30422061

[29] Magistro, D. et al. Measurement invariance of TGMD-3 in children with and without mental and behavioral disorders. *Psychol. Assess.* **30**, 1421 (2018). 29792504 10.1037/pas0000587

[30] Magistro, D. et al. Psychometric proprieties of the Test of Gross Motor Development-Third Edition in a large sample of Italian children. *J. Sci. Med. Sport* **23**, 860–865 (2020). 32146084 10.1016/j.jsams.2020.02.014

[31] Webster, E. K. & Ulrich, D. A. Evaluation of the psychometric properties of the Test of Gross Motor Development—Third edition. *J. Mot. Learn. Dev.* **5**, 45–58 (2017).

[32] Sim, J. & Wright, C. C. The Kappa statistic in reliability studies: Use, interpretation, and sample size requirements. *Phys. Ther.* **85**, 257–268 (2005). 15733050

[33] Maree, K. *Erikson’s Psychosocial Stages of Human Development*. (StatPearls Publishing).

[34] Eriksson, E. *Childhood and Society.* (W W Norton & Co., 1950).

[35] Centres for Disease Control and Prevention (CDC). Child Development: Milestones. (2023).

[36] Kellermann, T. S., Bonilha, L., Lin, J. J. & Hermann, B. P. Mapping the landscape of cognitive development in children with epilepsy. *Cortex* **66**, 1–8 (2015). 25776901 10.1016/j.cortex.2015.02.001 PMC4405468

[37] Shirinivas, S. G., Vetrivel, S. & Elango, N. M. Applications of graph theory in computer science an overview. *Int. J. Eng. Sci. Technol.* **2**, 4610–4621 (2010).

[38] Epskamp, S., Borsboom, D. & Fried, E. I. Estimating psychological networks and their accuracy: A tutorial paper. *Behav. Res. Methods* **50**, 195–212 (2018). 28342071 10.3758/s13428-017-0862-1 PMC5809547

[39] Friedman, J., Hastie, T. & Tibshirani, R. Sparse inverse covariance estimation with the graphical lasso. *Biostat. Oxf. Engl.* **9**, 432–441 (2008). 10.1093/biostatistics/kxm045 PMC3019769 18079126

[40] Epskamp, S. & Fried, E. I. A tutorial on regularized partial correlation networks. *Psychol. Methods* **23**, 617 (2018). 29595293 10.1037/met0000167

[41] McNeish, D. M. Using lasso for predictor selection and to assuage overfitting: A method long overlooked in behavioral sciences. *Multivar. Behav. Res.* **50**, 471–484 (2015). 10.1080/00273171.2015.1036965 26610247

[42] Chen, J. & Chen, Z. Extended Bayesian information criteria for model selection with large model spaces. *Biometrika* **95**, 759–771 (2008).

[43] Epskamp, S. Regularized Gaussian psychological networks: Brief report on the performance of extended BIC model selection. *ArXiv Prepr. ArXiv160605771* (2016).

[44] Busiello, D. M., Suweis, S., Hidalgo, J. & Maritan, A. Explorability and the origin of network sparsity in living systems. *Sci. Rep.* **7**, 12323 (2017). 28951597 10.1038/s41598-017-12521-1 PMC5615038

[45] Jones, P. J., Ma, R. & McNally, R. J. Bridge centrality: A network approach to understanding comorbidity. *Multivar. Behav. Res.* **56**, 353–367 (2021). 10.1080/00273171.2019.1614898 31179765

[46] Vagnetti, R. et al. Exploring the social cognition network in young adults with autism spectrum disorder using graph analysis. *Brain Behav.* **10**, e01524 (2020). 31971664 10.1002/brb3.1524 PMC7066354

[47] Boccaletti, S., Latora, V., Moreno, Y., Chavez, M. & Hwang, D.-U. Complex networks: Structure and dynamics. *Phys. Rep.* **424**, 175–308 (2006).

[48] Opsahl, T., Agneessens, F. & Skvoretz, J. Node centrality in weighted networks: Generalizing degree and shortest paths. *Soc. Netw.* **32**, 245–251 (2010).

[49] Dijkstra, E. W. A note on two problems in connexion with graphs. *Numer. Math.* **1**, 269–271 (1959).

[50] Team, R. C. R: A Language and Environment for Statistical Computing. (2020).

[51] Epskamp, S. *et al.* Package ‘qgraph’. (2017).

[52] Epskamp, S. & Fried, E. I. Package ‘bootnet’. *Bootstrap Methods Var. Netw. Estim. Routines* **5**, 0–1 (2015).

[53] Peper, L., Bootsma, R. J., Mestre, D. R. & Bakker, F. C. Catching balls: How to get the hand to the right place at the right time. *J. Exp. Psychol. Hum. Percept. Perform.* **20**, 591–612 (1994). 8027714 10.1037//0096-1523.20.3.591

[54] Van Waelvelde, H., De Weerdt, W., De Cock, P. & Engelsman, B. C. M. S. Ball catching. Can it be measured? *Physiother. Theory Pract.* **19**, 259–267 (2003).

[55] Ashby, B. M. & Heegaard, J. H. Role of arm motion in the standing long jump. *J. Biomech.* **35**, 1631–1637 (2002). 12445616 10.1016/s0021-9290(02)00239-7

[56] Whitall, J. & Getchell, N. From walking to running: Applying a dynamical systems approach to the development of locomotor skills. *Child Dev.* **66**, 1541–1553 (1995). 7555229

[57] Forssberg, H. Neural control of human motor development. *Curr. Opin. Neurobiol.* **9**, 676–682 (1999). 10607646 10.1016/s0959-4388(99)00037-9

[58] Lacquaniti, F., Ivanenko, Y. P. & Zago, M. Development of human locomotion. *Curr. Opin. Neurobiol.* **22**, 822–828 (2012). 22498713 10.1016/j.conb.2012.03.012

[59] Beach, P. S., Perreault, M., Brian, A. & Collier, D. H. *Motor Learning and Development* (Human Kinetics, 2023).

[60] Gerber, R. J., Wilks, T. & Erdie-Lalena, C. Developmental milestones: motor development. *Pediatr. Rev.* **31**, 267–276; quiz 277 (2010). 10.1542/pir.31-7-267 20595440

[61] Thach, W. T. A role for the cerebellum in learning movement coordination. *Neurobiol. Learn. Mem.* **70**, 177–188 (1998). 9753595 10.1006/nlme.1998.3846

