# Supplementary material for: Mapping relationships among gross motor skills in 16,989 children using network analysis
Source: Sci Rep. 2025 Apr 4;15:11591. doi: 10.1038/s41598-025-95924-9 (PMC11971267; doi:10.1038/s41598-025-95924-9)
Supplement: Supplementary file 1 — Supplementary Material 1 [file 41598_2025_95924_MOESM1_ESM.docx]

MAPPING RELATIONSHIPS AMONG GROSS MOTOR SKILLS IN 16,989 CHILDREN USING NETWORK ANALYSIS

Roberto Vagnetti^1,4^, Simon Cooper^1^, Fabio Carlevaro^2^, Ruth Boat^1^, Francesca Magno^2,3^, Giovanni Musella^2,3^, Daniele Magistro^1*^

^1^ Department of Sport Science, School of Science and Technology, Nottingham Trent University, United Kingdom

^2^ Polo Universitario Asti Studi Superiori (Uni-Astiss), Italy

^3^ Dipartimento di Scienze della Vita e Biologia dei Sistemi, University of Torino, Italy

^4^ School of Environment, Education and Development, University of Manchester, United Kingdom

*Corresponding author:

Daniele Magistro, Ph.D.

Department of Sport Science

School of Science and Technology

Nottingham Trent University

College Drive

Clifton

Nottingham

NG11 8NS

United Kingdom

Phone: +44 (0)115 848 3522

Email: [daniele.magistro@ntu.ac.uk](mailto:daniele.magistro@ntu.ac.uk)

Author contact details:

Roberto Vagnetti, Ph.D.

School of Environment, Education and Development, University of Manchester, Manchester, M13 9PL, United Kingdom

Email: roberto.vagnetti@manchester.ac.uk

Simon Cooper, Ph.D.

Department of Sport Science, School of Science and Technology, Nottingham Trent University, College Drive, Clifton, Nottingham, NG11 8NS, United Kingdom

Email: simon.cooper@ntu.ac.uk

Fabio Carlevaro, Ph.D.

Polo Universitario Asti Studi Superiori (Uni-Astiss), Area Fabrizio De Andrè, 14100 Asti, Italy

Email: fabio.carlevaro@unito.it

Ruth Boat, Ph.D.

Department of Sport Science, School of Science and Technology, Nottingham Trent University, College Drive, Clifton, Nottingham, NG11 8NS, United Kingdom

Email: ruth.boat@ntu.ac.uk

Francesca Magno, Ph.D.

Polo Universitario Asti Studi Superiori (Uni-Astiss), Area Fabrizio De Andrè, 14100 Asti, Italy

Email: francesca.magno@unito.it

Giovanni Musella, Ph.D.

Polo Universitario Asti Studi Superiori (Uni-Astiss), Area Fabrizio De Andrè, 14100 Asti, Italy

Email: giovanni.musella@unito.it

*Supplementary material*

Figure S1. Networks of Locomotor skills (Ls) and Ball skills (Bs) with fixed node positions for the whole sample and age groups.


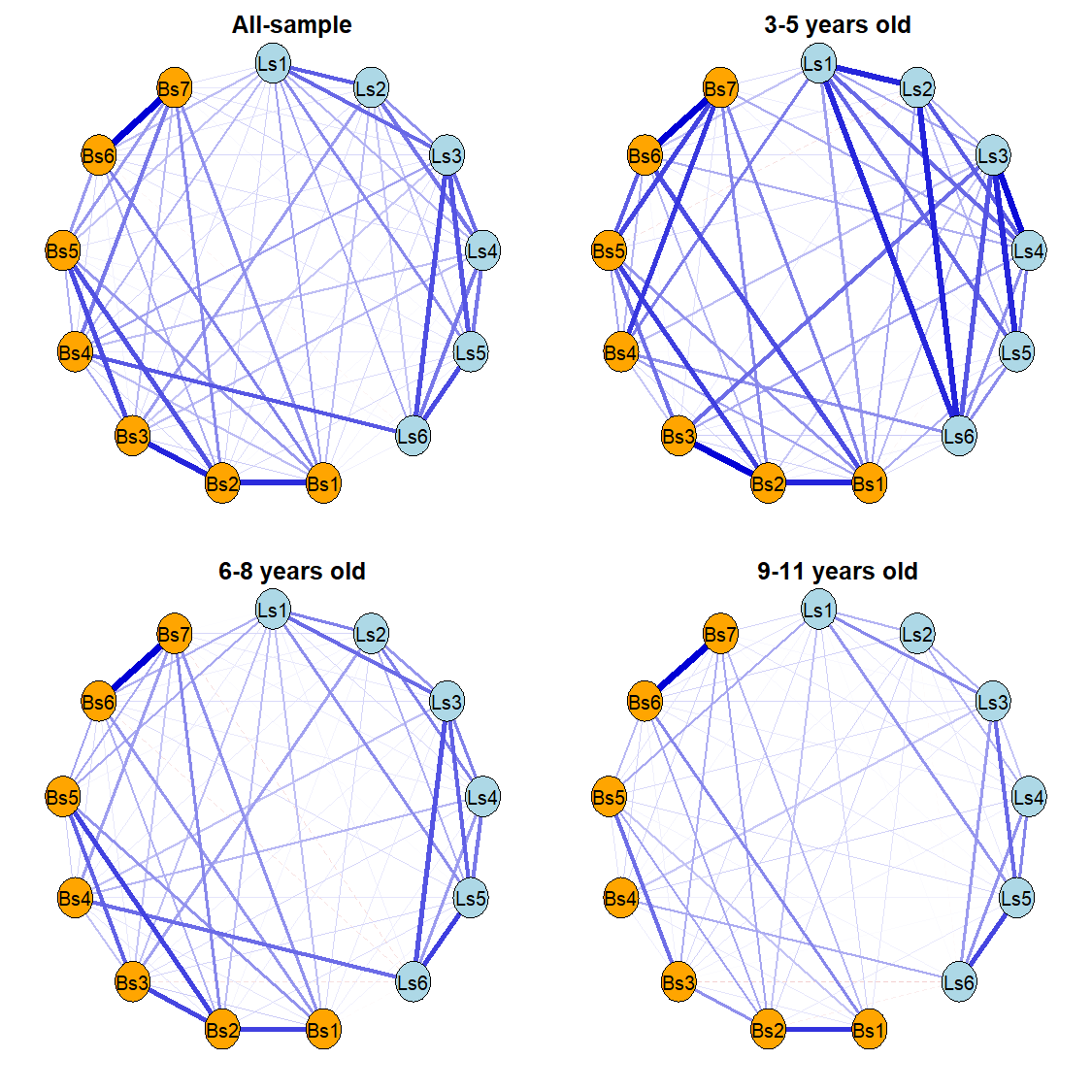


Figure S2. Graphical representations of networks of Locomotor skills (Ls) and Ball skills (Bs) for each age group, according to sex.


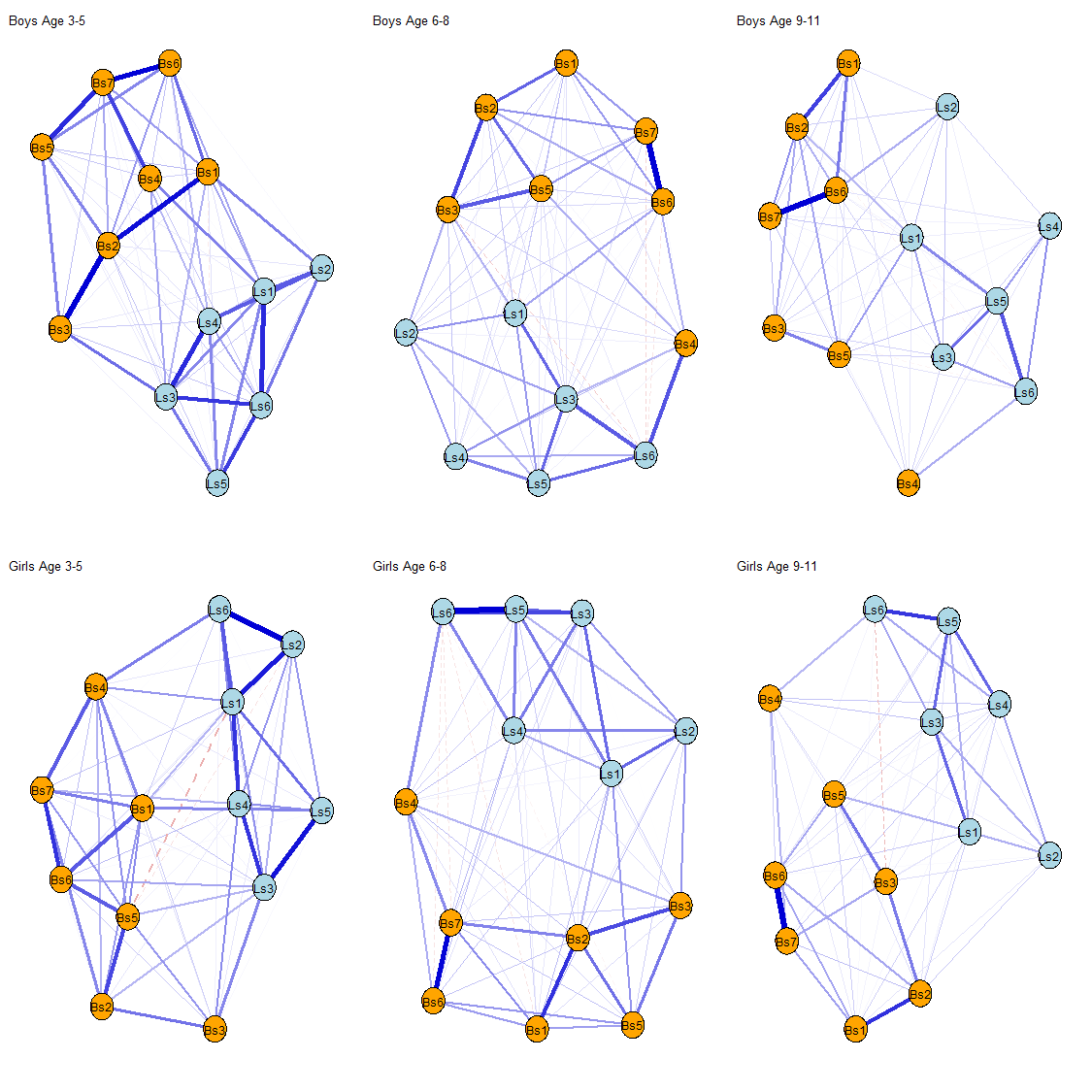


Figure S3. Networks with fixed node positions for each age group, according to sex.


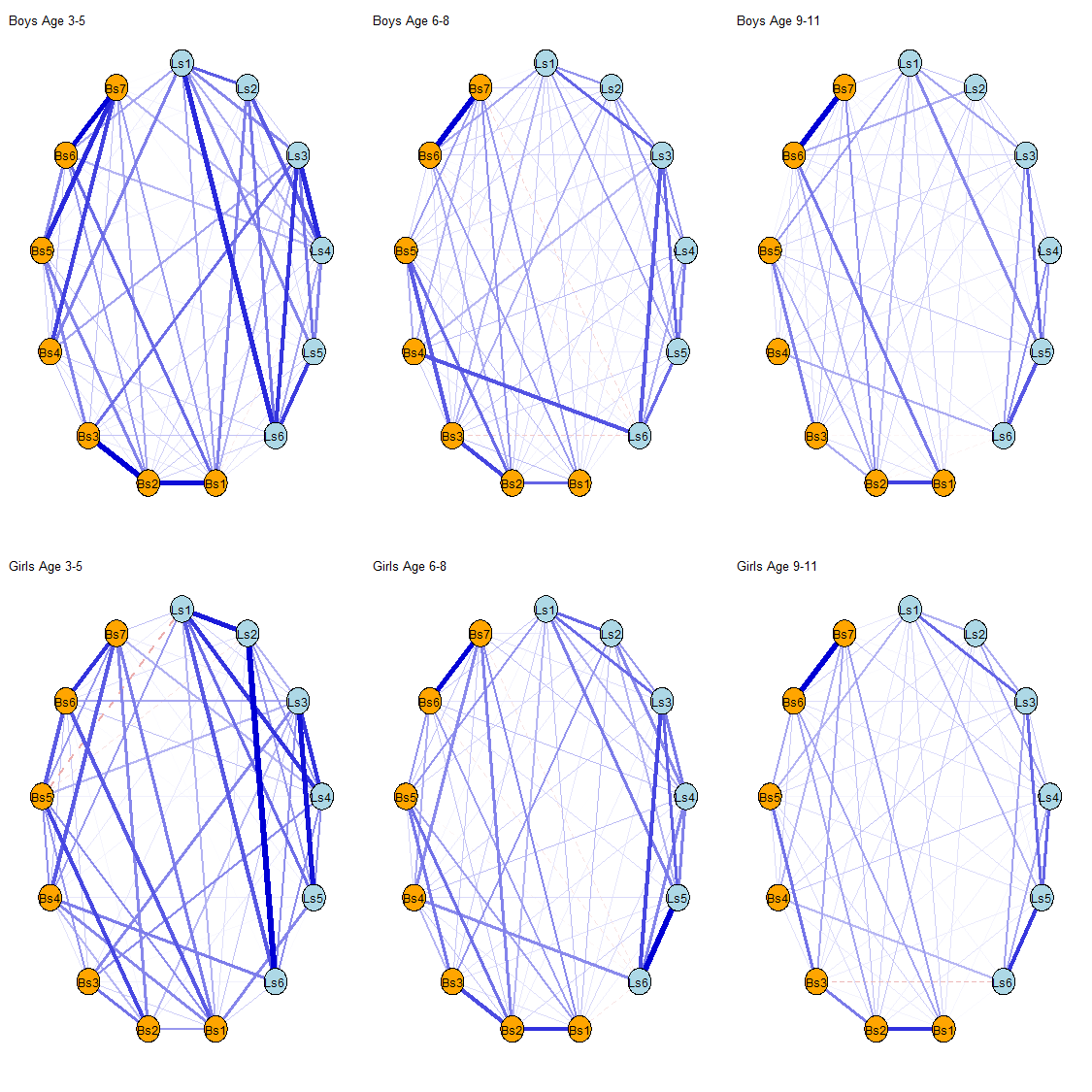


Figure S4. Boys’ centrality statistics for each age strata.


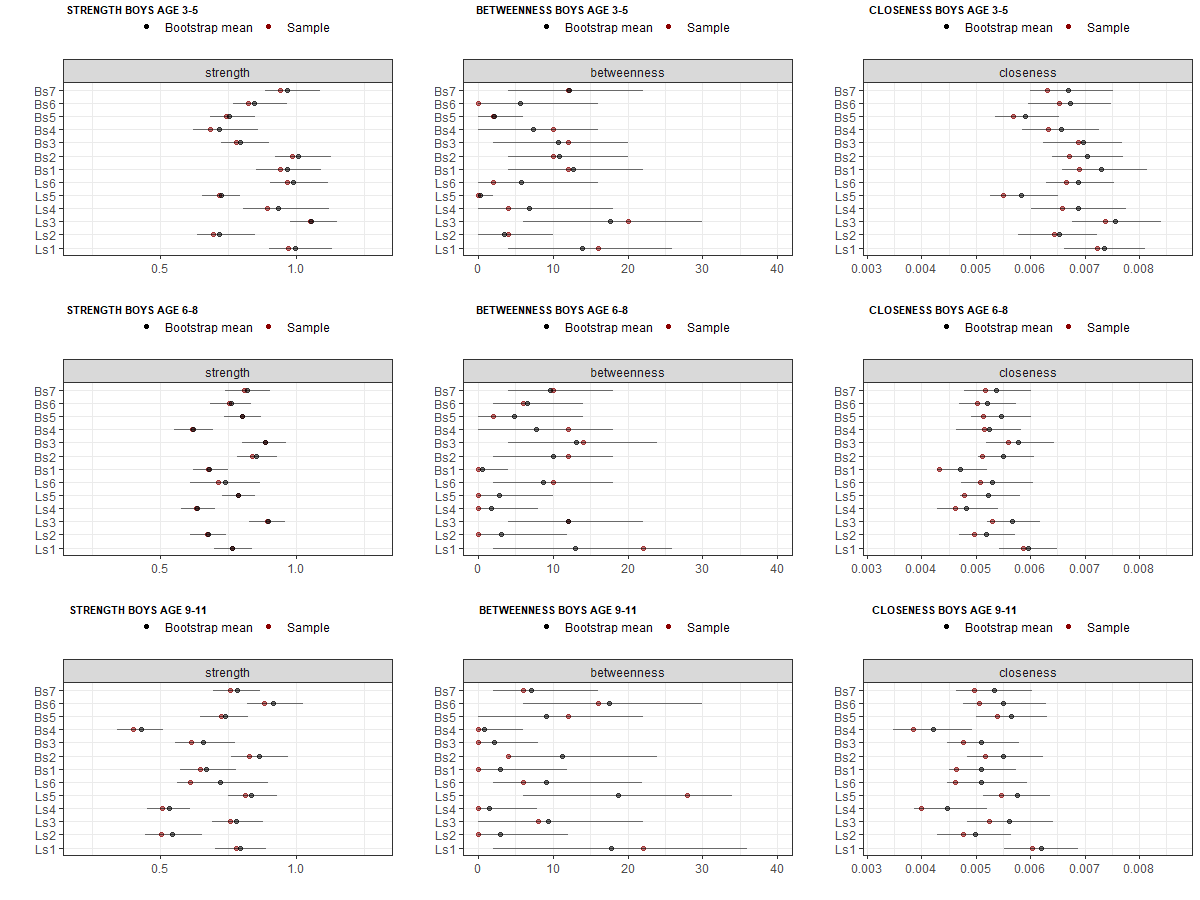


Figure S5. Boys’ bridge centrality statistics for each age strata.


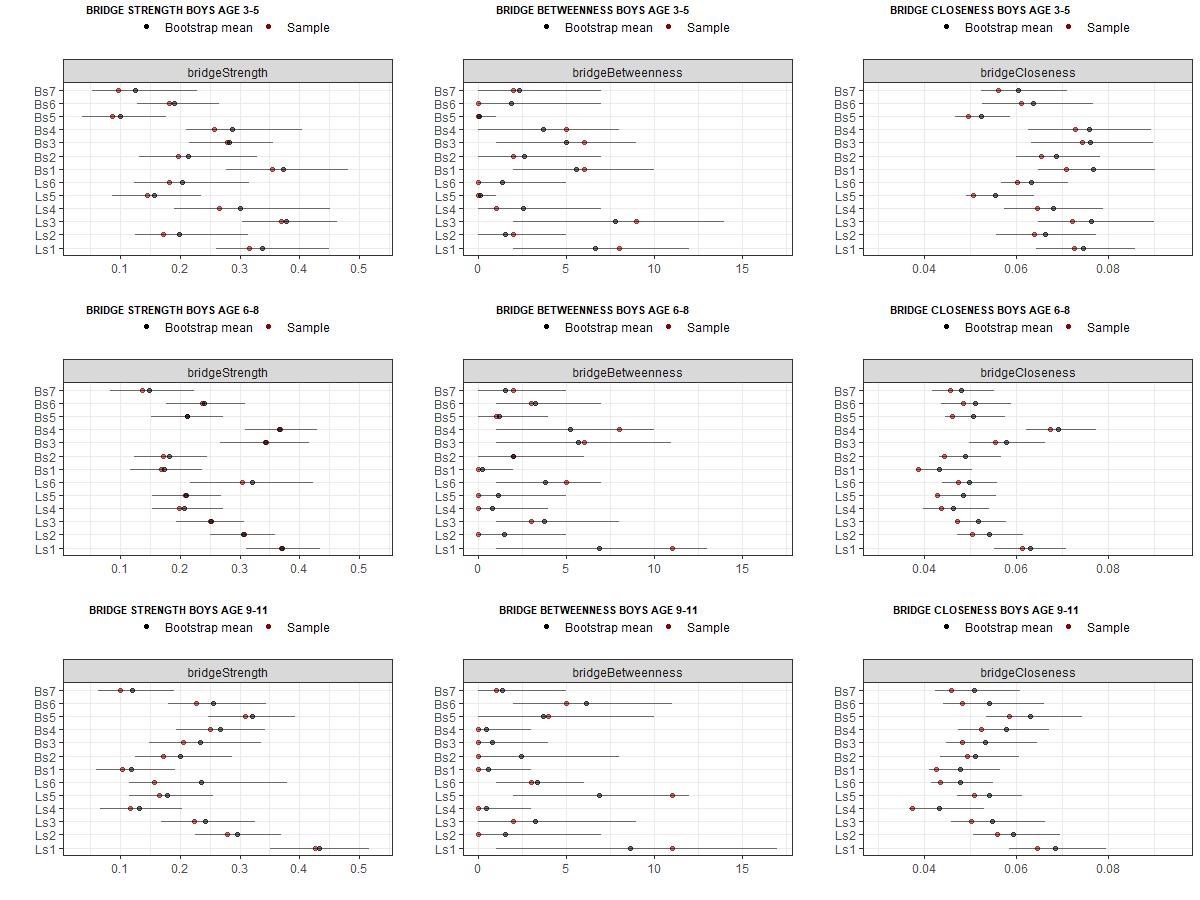


Figure S6. Girls’ centrality statistics for each age strata.


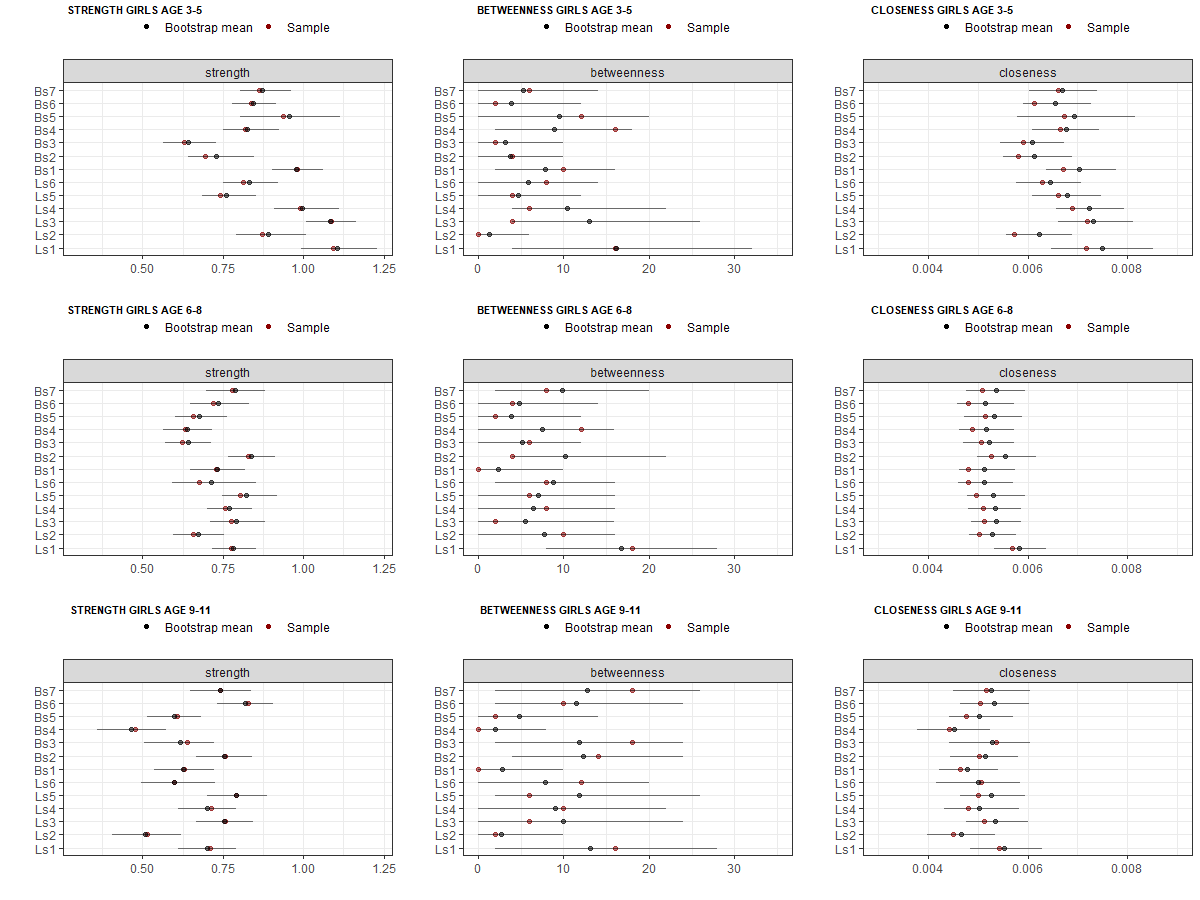


Figure S7. Girls’ bridge centrality statistics for each age strata.


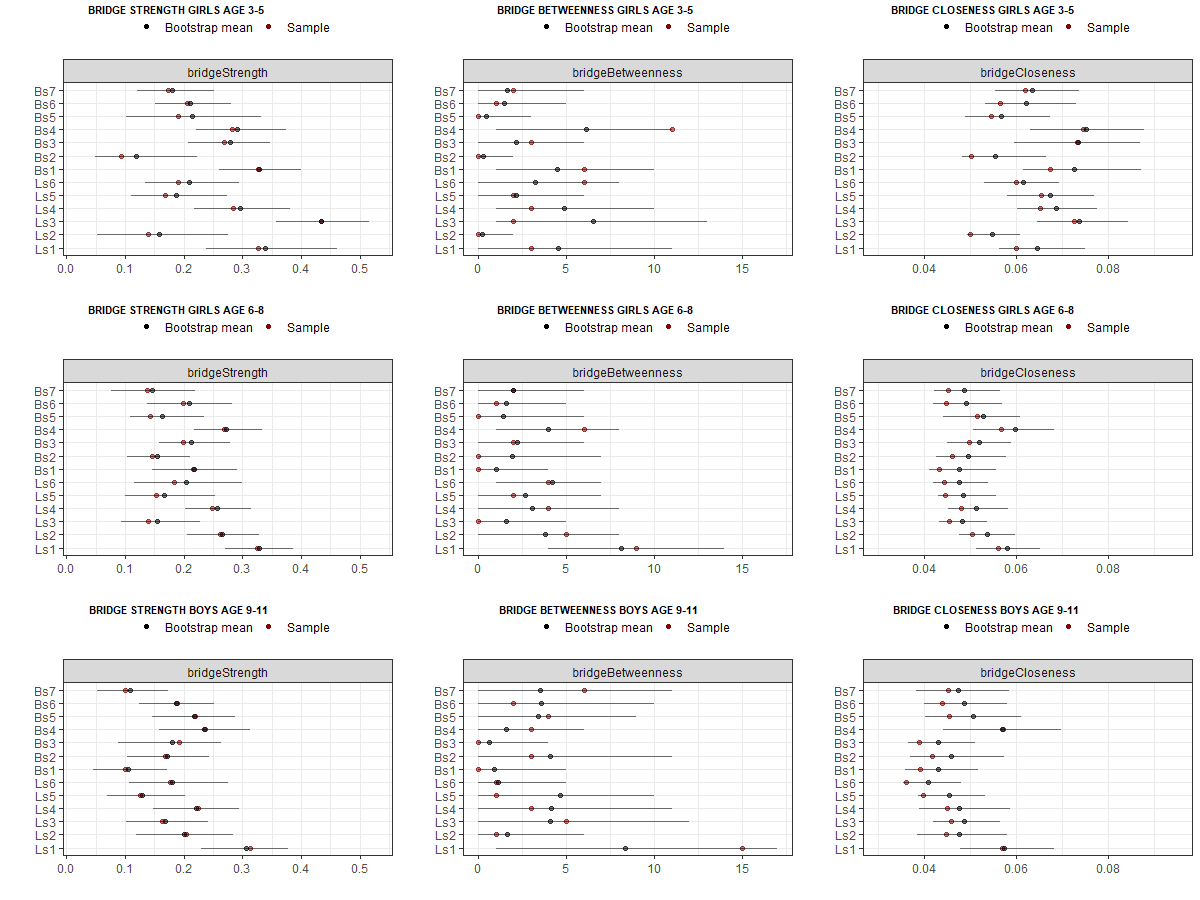


Table S1. Regularised partial correlations weights for the all-sample network.

|  | Ls1 | Ls2 | Ls3 | Ls4 | Ls5 | Ls6 | Bs1 | Bs2 | Bs3 | Bs4 | Bs5 | Bs6 | Bs7 |
| --- | --- | --- | --- | --- | --- | --- | --- | --- | --- | --- | --- | --- | --- |
| Ls1 | 0 | 0.159 | 0.148 | 0.074 | 0.115 | 0.089 | 0.091 | 0.048 | 0.037 | 0.072 | 0.06 | 0.065 | 0.033 |
| Ls2 | 0.159 | 0 | 0.108 | 0.136 | 0.064 | 0.057 | 0.066 | 0.035 | 0.097 | 0.044 | 0 | 0.028 | 0.025 |
| Ls3 | 0.148 | 0.108 | 0 | 0.145 | 0.167 | 0.174 | 0.039 | 0.034 | 0.056 | 0.091 | 0.03 | 0.04 | 0.029 |
| Ls4 | 0.074 | 0.136 | 0.145 | 0 | 0.133 | 0.133 | 0.01 | 0.007 | 0.066 | 0.064 | 0 | 0.037 | 0.049 |
| Ls5 | 0.115 | 0.064 | 0.167 | 0.133 | 0 | 0.178 | 0.019 | 0.039 | 0.035 | 0.024 | 0.014 | 0.029 | 0.009 |
| Ls6 | 0.089 | 0.057 | 0.174 | 0.133 | 0.178 | 0 | 0.014 | 0 | 0 | 0.165 | 0.014 | -0.012 | 0 |
| Bs1 | 0.091 | 0.066 | 0.039 | 0.01 | 0.019 | 0.014 | 0 | 0.208 | 0.05 | 0.065 | 0.1 | 0.125 | 0.109 |
| Bs2 | 0.048 | 0.035 | 0.034 | 0.007 | 0.039 | 0 | 0.208 | 0 | 0.214 | 0.046 | 0.17 | 0.081 | 0.114 |
| Bs3 | 0.037 | 0.097 | 0.056 | 0.066 | 0.035 | 0 | 0.05 | 0.214 | 0 | 0.086 | 0.176 | 0.013 | 0.07 |
| Bs4 | 0.072 | 0.044 | 0.091 | 0.064 | 0.024 | 0.165 | 0.065 | 0.046 | 0.086 | 0 | 0.078 | 0.044 | 0.131 |
| Bs5 | 0.06 | 0 | 0.03 | 0 | 0.014 | 0.014 | 0.1 | 0.17 | 0.176 | 0.078 | 0 | 0.099 | 0.101 |
| Bs6 | 0.065 | 0.028 | 0.04 | 0.037 | 0.029 | -0.012 | 0.125 | 0.081 | 0.013 | 0.044 | 0.099 | 0 | 0.25 |
| Bs7 | 0.033 | 0.025 | 0.029 | 0.049 | 0.009 | 0 | 0.109 | 0.114 | 0.07 | 0.131 | 0.101 | 0.25 | 0 |

Table S2. Regularised partial correlations weights for the 3-5 years old group.

|  | Ls1 | Ls2 | Ls3 | Ls4 | Ls5 | Ls6 | Bs1 | Bs2 | Bs3 | Bs4 | Bs5 | Bs6 | Bs7 |
| --- | --- | --- | --- | --- | --- | --- | --- | --- | --- | --- | --- | --- | --- |
| Ls1 | 0 | 0.184 | 0.078 | 0.128 | 0.131 | 0.184 | 0.082 | 0 | 0 | 0.111 | 0 | 0.052 | 0.033 |
| Ls2 | 0.184 | 0 | 0.069 | 0.138 | 0.062 | 0.188 | 0.078 | 0 | 0.02 | 0 | -0.026 | 0.023 | 0 |
| Ls3 | 0.078 | 0.069 | 0 | 0.207 | 0.184 | 0.144 | 0.06 | 0.049 | 0.125 | 0.056 | 0.03 | 0.03 | 0.017 |
| Ls4 | 0.128 | 0.138 | 0.207 | 0 | 0.111 | 0.097 | -0.009 | 0.012 | 0.073 | 0 | 0 | 0.07 | 0.071 |
| Ls5 | 0.131 | 0.062 | 0.184 | 0.111 | 0 | 0.097 | 0.069 | 0.023 | 0.019 | 0.003 | 0 | 0.021 | 0.017 |
| Ls6 | 0.184 | 0.188 | 0.144 | 0.097 | 0.097 | 0 | 0.034 | 0.014 | 0.043 | 0.095 | 0 | 0 | 0 |
| Bs1 | 0.082 | 0.078 | 0.06 | -0.009 | 0.069 | 0.034 | 0 | 0.187 | 0.04 | 0.078 | 0.093 | 0.152 | 0.106 |
| Bs2 | 0 | 0 | 0.049 | 0.012 | 0.023 | 0.014 | 0.187 | 0 | 0.214 | 0.041 | 0.164 | 0.065 | 0.102 |
| Bs3 | 0 | 0.02 | 0.125 | 0.073 | 0.019 | 0.043 | 0.04 | 0.214 | 0 | 0.049 | 0.121 | 0.007 | 0.025 |
| Bs4 | 0.111 | 0 | 0.056 | 0 | 0.003 | 0.095 | 0.078 | 0.041 | 0.049 | 0 | 0.075 | 0.063 | 0.17 |
| Bs5 | 0 | -0.026 | 0.03 | 0 | 0 | 0 | 0.093 | 0.164 | 0.121 | 0.075 | 0 | 0.139 | 0.159 |
| Bs6 | 0.052 | 0.023 | 0.03 | 0.07 | 0.021 | 0 | 0.152 | 0.065 | 0.007 | 0.063 | 0.139 | 0 | 0.216 |
| Bs7 | 0.033 | 0 | 0.017 | 0.071 | 0.017 | 0 | 0.106 | 0.102 | 0.025 | 0.17 | 0.159 | 0.216 | 0 |

Table S3. Regularised partial correlations weights for the 6-8 years old group.

|  | Ls1 | Ls2 | Ls3 | Ls4 | Ls5 | Ls6 | Bs1 | Bs2 | Bs3 | Bs4 | Bs5 | Bs6 | Bs7 |
| --- | --- | --- | --- | --- | --- | --- | --- | --- | --- | --- | --- | --- | --- |
| Ls1 | 0 | 0.118 | 0.14 | 0.039 | 0.105 | 0.014 | 0.06 | 0.062 | 0.061 | 0.022 | 0.08 | 0.075 | 0.002 |
| Ls2 | 0.118 | 0 | 0.099 | 0.12 | 0.078 | 0 | 0.039 | 0.011 | 0.088 | 0.022 | 0.011 | 0.015 | 0.039 |
| Ls3 | 0.14 | 0.099 | 0 | 0.118 | 0.147 | 0.16 | 0.021 | 0.018 | 0.018 | 0.059 | 0 | 0.035 | 0.022 |
| Ls4 | 0.039 | 0.12 | 0.118 | 0 | 0.125 | 0.094 | 0.017 | 0 | 0.039 | 0.068 | 0 | 0.02 | 0.019 |
| Ls5 | 0.105 | 0.078 | 0.147 | 0.125 | 0 | 0.18 | 0.013 | 0.034 | 0.04 | 0.025 | 0 | 0.034 | 0.005 |
| Ls6 | 0.014 | 0 | 0.16 | 0.094 | 0.18 | 0 | -0.005 | 0 | -0.029 | 0.138 | 0 | -0.028 | -0.031 |
| Bs1 | 0.06 | 0.039 | 0.021 | 0.017 | 0.013 | -0.005 | 0 | 0.178 | 0.049 | 0.027 | 0.102 | 0.098 | 0.103 |
| Bs2 | 0.062 | 0.011 | 0.018 | 0 | 0.034 | 0 | 0.178 | 0 | 0.173 | 0.029 | 0.177 | 0.074 | 0.111 |
| Bs3 | 0.061 | 0.088 | 0.018 | 0.039 | 0.04 | -0.029 | 0.049 | 0.173 | 0 | 0.059 | 0.148 | 0.021 | 0.053 |
| Bs4 | 0.022 | 0.022 | 0.059 | 0.068 | 0.025 | 0.138 | 0.027 | 0.029 | 0.059 | 0 | 0.049 | 0.036 | 0.091 |
| Bs5 | 0.08 | 0.011 | 0 | 0 | 0 | 0 | 0.102 | 0.177 | 0.148 | 0.049 | 0 | 0.086 | 0.081 |
| Bs6 | 0.075 | 0.015 | 0.035 | 0.02 | 0.034 | -0.028 | 0.098 | 0.074 | 0.021 | 0.036 | 0.086 | 0 | 0.239 |
| Bs7 | 0.002 | 0.039 | 0.022 | 0.019 | 0.005 | -0.031 | 0.103 | 0.111 | 0.053 | 0.091 | 0.081 | 0.239 | 0 |

Table S4. Regularised partial correlations weights for the 9-11 years old group.

|  | Ls1 | Ls2 | Ls3 | Ls4 | Ls5 | Ls6 | Bs1 | Bs2 | Bs3 | Bs4 | Bs5 | Bs6 | Bs7 |
| --- | --- | --- | --- | --- | --- | --- | --- | --- | --- | --- | --- | --- | --- |
| Ls1 | 0 | 0.088 | 0.139 | 0.034 | 0.123 | 0.005 | 0.056 | 0.071 | 0.045 | 0.013 | 0.101 | 0.036 | 0.05 |
| Ls2 | 0.088 | 0 | 0.092 | 0.091 | 0.026 | -0.004 | 0.032 | 0.052 | 0.054 | 0.019 | 0 | 0.052 | 0 |
| Ls3 | 0.139 | 0.092 | 0 | 0.066 | 0.173 | 0.115 | 0 | 0 | 0.031 | 0.065 | 0.043 | 0.049 | 0.009 |
| Ls4 | 0.034 | 0.091 | 0.066 | 0 | 0.142 | 0.127 | 0 | 0 | 0.02 | 0.054 | 0 | 0.026 | 0.035 |
| Ls5 | 0.123 | 0.026 | 0.173 | 0.142 | 0 | 0.216 | 0 | 0.027 | 0.005 | 0.018 | 0.048 | 0.03 | 0.008 |
| Ls6 | 0.005 | -0.004 | 0.115 | 0.127 | 0.216 | 0 | 0 | -0.021 | -0.055 | 0.098 | 0.015 | 0 | 0 |
| Bs1 | 0.056 | 0.032 | 0 | 0 | 0 | 0 | 0 | 0.239 | 0.031 | 0.028 | 0.07 | 0.139 | 0.085 |
| Bs2 | 0.071 | 0.052 | 0 | 0 | 0.027 | -0.021 | 0.239 | 0 | 0.129 | 0 | 0.108 | 0.103 | 0.105 |
| Bs3 | 0.045 | 0.054 | 0.031 | 0.02 | 0.005 | -0.055 | 0.031 | 0.129 | 0 | 0.021 | 0.169 | 0.018 | 0.094 |
| Bs4 | 0.013 | 0.019 | 0.065 | 0.054 | 0.018 | 0.098 | 0.028 | 0 | 0.021 | 0 | 0.032 | 0.051 | 0.054 |
| Bs5 | 0.101 | 0 | 0.043 | 0 | 0.048 | 0.015 | 0.07 | 0.108 | 0.169 | 0.032 | 0 | 0.082 | 0.035 |
| Bs6 | 0.036 | 0.052 | 0.049 | 0.026 | 0.03 | 0 | 0.139 | 0.103 | 0.018 | 0.051 | 0.082 | 0 | 0.297 |
| Bs7 | 0.05 | 0 | 0.009 | 0.035 | 0.008 | 0 | 0.085 | 0.105 | 0.094 | 0.054 | 0.035 | 0.297 | 0 |

Table S5. Regularised partial correlations weights for boys in the 3-5 years old group.

|  | Ls1 | Ls2 | Ls3 | Ls4 | Ls5 | Ls6 | Bs1 | Bs2 | Bs3 | Bs4 | Bs5 | Bs6 | Bs7 |
| --- | --- | --- | --- | --- | --- | --- | --- | --- | --- | --- | --- | --- | --- |
| Ls1 | 0 | 0.151 | 0.113 | 0.078 | 0.114 | 0.2 | 0.089 | 0 | 0 | 0.119 | 0.019 | 0.085 | 0.003 |
| Ls2 | 0.151 | 0 | 0.054 | 0.154 | 0.035 | 0.128 | 0.118 | 0.024 | 0.02 | 0 | 0 | 0.007 | 0.003 |
| Ls3 | 0.113 | 0.054 | 0 | 0.199 | 0.128 | 0.188 | 0.073 | 0.044 | 0.136 | 0.085 | 0.018 | 0 | 0.014 |
| Ls4 | 0.078 | 0.154 | 0.199 | 0 | 0.113 | 0.086 | -0.019 | 0.028 | 0.048 | 0 | 0.03 | 0.07 | 0.07 |
| Ls5 | 0.114 | 0.035 | 0.128 | 0.113 | 0 | 0.182 | 0.028 | 0.054 | 0.017 | 0 | 0.019 | 0.019 | 0.007 |
| Ls6 | 0.2 | 0.128 | 0.188 | 0.086 | 0.182 | 0 | 0.027 | 0.046 | 0.057 | 0.053 | 0 | 0 | 0 |
| Bs1 | 0.089 | 0.118 | 0.073 | -0.019 | 0.028 | 0.027 | 0 | 0.227 | 0.023 | 0.045 | 0.063 | 0.139 | 0.093 |
| Bs2 | 0 | 0.024 | 0.044 | 0.028 | 0.054 | 0.046 | 0.227 | 0 | 0.239 | 0.028 | 0.117 | 0.098 | 0.082 |
| Bs3 | 0 | 0.02 | 0.136 | 0.048 | 0.017 | 0.057 | 0.023 | 0.239 | 0 | 0.066 | 0.115 | 0 | 0.06 |
| Bs4 | 0.119 | 0 | 0.085 | 0 | 0 | 0.053 | 0.045 | 0.028 | 0.066 | 0 | 0.053 | 0.059 | 0.177 |
| Bs5 | 0.019 | 0 | 0.018 | 0.03 | 0.019 | 0 | 0.063 | 0.117 | 0.115 | 0.053 | 0 | 0.113 | 0.199 |
| Bs6 | 0.085 | 0.007 | 0 | 0.07 | 0.019 | 0 | 0.139 | 0.098 | 0 | 0.059 | 0.113 | 0 | 0.235 |
| Bs7 | 0.003 | 0.003 | 0.014 | 0.07 | 0.007 | 0 | 0.093 | 0.082 | 0.06 | 0.177 | 0.199 | 0.235 | 0 |

Table S6. Regularised partial correlations weights for boys in the 6-8 years old group.

|  | Ls1 | Ls2 | Ls3 | Ls4 | Ls5 | Ls6 | Bs1 | Bs2 | Bs3 | Bs4 | Bs5 | Bs6 | Bs7 |
| --- | --- | --- | --- | --- | --- | --- | --- | --- | --- | --- | --- | --- | --- |
| Ls1 | 0 | 0.103 | 0.147 | 0.022 | 0.098 | 0.026 | 0.054 | 0.056 | 0.075 | 0.035 | 0.054 | 0.087 | 0.008 |
| Ls2 | 0.103 | 0 | 0.09 | 0.102 | 0.071 | 0 | 0.034 | 0.031 | 0.08 | 0.015 | 0.054 | 0.043 | 0.049 |
| Ls3 | 0.147 | 0.09 | 0 | 0.104 | 0.147 | 0.157 | 0.03 | 0.037 | 0.029 | 0.069 | 0.035 | 0.046 | 0.005 |
| Ls4 | 0.022 | 0.102 | 0.104 | 0 | 0.122 | 0.084 | 0.032 | 0.011 | 0.061 | 0.067 | 0.022 | 0.006 | 0 |
| Ls5 | 0.098 | 0.071 | 0.147 | 0.122 | 0 | 0.143 | 0.019 | 0.037 | 0.047 | 0.016 | 0.026 | 0.029 | 0.034 |
| Ls6 | 0.026 | 0 | 0.157 | 0.084 | 0.143 | 0 | 0 | 0 | -0.052 | 0.165 | 0.022 | -0.026 | -0.04 |
| Bs1 | 0.054 | 0.034 | 0.03 | 0.032 | 0.019 | 0 | 0 | 0.15 | 0.064 | 0.03 | 0.068 | 0.1 | 0.098 |
| Bs2 | 0.056 | 0.031 | 0.037 | 0.011 | 0.037 | 0 | 0.15 | 0 | 0.179 | 0.01 | 0.143 | 0.081 | 0.105 |
| Bs3 | 0.075 | 0.08 | 0.029 | 0.061 | 0.047 | -0.052 | 0.064 | 0.179 | 0 | 0.054 | 0.159 | 0.039 | 0.05 |
| Bs4 | 0.035 | 0.015 | 0.069 | 0.067 | 0.016 | 0.165 | 0.03 | 0.01 | 0.054 | 0 | 0.077 | 0.006 | 0.076 |
| Bs5 | 0.054 | 0.054 | 0.035 | 0.022 | 0.026 | 0.022 | 0.068 | 0.143 | 0.159 | 0.077 | 0 | 0.046 | 0.098 |
| Bs6 | 0.087 | 0.043 | 0.046 | 0.006 | 0.029 | -0.026 | 0.1 | 0.081 | 0.039 | 0.006 | 0.046 | 0 | 0.246 |
| Bs7 | 0.008 | 0.049 | 0.005 | 0 | 0.034 | -0.04 | 0.098 | 0.105 | 0.05 | 0.076 | 0.098 | 0.246 | 0 |

Table S7. Regularised partial correlations weights for boys in the 9-11 years old group.

|  | Ls1 | Ls2 | Ls3 | Ls4 | Ls5 | Ls6 | Bs1 | Bs2 | Bs3 | Bs4 | Bs5 | Bs6 | Bs7 |
| --- | --- | --- | --- | --- | --- | --- | --- | --- | --- | --- | --- | --- | --- |
| Ls1 | 0 | 0.077 | 0.106 | 0.02 | 0.14 | 0.012 | 0.061 | 0.084 | 0.057 | 0.028 | 0.116 | 0.014 | 0.065 |
| Ls2 | 0.077 | 0 | 0.077 | 0.055 | 0.018 | 0 | 0.042 | 0.057 | 0.066 | 0 | 0.017 | 0.097 | 0 |
| Ls3 | 0.106 | 0.077 | 0 | 0.063 | 0.175 | 0.113 | 0 | 0 | 0.053 | 0.055 | 0.056 | 0.056 | 0.004 |
| Ls4 | 0.02 | 0.055 | 0.063 | 0 | 0.118 | 0.135 | 0 | 0 | 0.018 | 0.032 | 0.014 | 0.036 | 0.016 |
| Ls5 | 0.14 | 0.018 | 0.175 | 0.118 | 0 | 0.196 | 0 | 0.015 | 0 | 0.039 | 0.073 | 0.024 | 0.014 |
| Ls6 | 0.012 | 0 | 0.113 | 0.135 | 0.196 | 0 | 0 | -0.016 | -0.011 | 0.097 | 0.033 | 0 | 0 |
| Bs1 | 0.061 | 0.042 | 0 | 0 | 0 | 0 | 0 | 0.224 | 0.042 | 0.032 | 0.028 | 0.158 | 0.059 |
| Bs2 | 0.084 | 0.057 | 0 | 0 | 0.015 | -0.016 | 0.224 | 0 | 0.1 | 0 | 0.107 | 0.094 | 0.13 |
| Bs3 | 0.057 | 0.066 | 0.053 | 0.018 | 0 | -0.011 | 0.042 | 0.1 | 0 | 0 | 0.149 | 0.038 | 0.083 |
| Bs4 | 0.028 | 0 | 0.055 | 0.032 | 0.039 | 0.097 | 0.032 | 0 | 0 | 0 | 0.049 | 0.05 | 0.018 |
| Bs5 | 0.116 | 0.017 | 0.056 | 0.014 | 0.073 | 0.033 | 0.028 | 0.107 | 0.149 | 0.049 | 0 | 0.014 | 0.069 |
| Bs6 | 0.014 | 0.097 | 0.056 | 0.036 | 0.024 | 0 | 0.158 | 0.094 | 0.038 | 0.05 | 0.014 | 0 | 0.3 |
| Bs7 | 0.065 | 0 | 0.004 | 0.016 | 0.014 | 0 | 0.059 | 0.13 | 0.083 | 0.018 | 0.069 | 0.3 | 0 |

Table S8. Regularised partial correlations weights for girls in the 3-5 years old group.

|  | Ls1 | Ls2 | Ls3 | Ls4 | Ls5 | Ls6 | Bs1 | Bs2 | Bs3 | Bs4 | Bs5 | Bs6 | Bs7 |
| --- | --- | --- | --- | --- | --- | --- | --- | --- | --- | --- | --- | --- | --- |
| Ls1 | 0 | 0.218 | 0.054 | 0.194 | 0.141 | 0.159 | 0.071 | 0 | 0 | 0.098 | -0.077 | 0.021 | 0.059 |
| Ls2 | 0.218 | 0 | 0.074 | 0.101 | 0.098 | 0.243 | 0.031 | 0 | 0.023 | 0.024 | -0.029 | 0.032 | 0 |
| Ls3 | 0.054 | 0.074 | 0 | 0.194 | 0.225 | 0.106 | 0.059 | 0.07 | 0.115 | 0.014 | 0.071 | 0.085 | 0.021 |
| Ls4 | 0.194 | 0.101 | 0.194 | 0 | 0.107 | 0.109 | 0 | 0.024 | 0.111 | 0 | 0.014 | 0.063 | 0.073 |
| Ls5 | 0.141 | 0.098 | 0.225 | 0.107 | 0 | 0.004 | 0.116 | 0 | 0.007 | 0.023 | 0 | 0.005 | 0.017 |
| Ls6 | 0.159 | 0.243 | 0.106 | 0.109 | 0.004 | 0 | 0.05 | 0 | 0.014 | 0.124 | 0 | 0 | 0.003 |
| Bs1 | 0.071 | 0.031 | 0.059 | 0 | 0.116 | 0.05 | 0 | 0.099 | 0.047 | 0.115 | 0.11 | 0.158 | 0.124 |
| Bs2 | 0 | 0 | 0.07 | 0.024 | 0 | 0 | 0.099 | 0 | 0.133 | 0.071 | 0.175 | 0.01 | 0.115 |
| Bs3 | 0 | 0.023 | 0.115 | 0.111 | 0.007 | 0.014 | 0.047 | 0.133 | 0 | 0.028 | 0.1 | 0.051 | 0 |
| Bs4 | 0.098 | 0.024 | 0.014 | 0 | 0.023 | 0.124 | 0.115 | 0.071 | 0.028 | 0 | 0.105 | 0.062 | 0.156 |
| Bs5 | -0.077 | -0.029 | 0.071 | 0.014 | 0 | 0 | 0.11 | 0.175 | 0.1 | 0.105 | 0 | 0.155 | 0.101 |
| Bs6 | 0.021 | 0.032 | 0.085 | 0.063 | 0.005 | 0 | 0.158 | 0.01 | 0.051 | 0.062 | 0.155 | 0 | 0.195 |
| Bs7 | 0.059 | 0 | 0.021 | 0.073 | 0.017 | 0.003 | 0.124 | 0.115 | 0 | 0.156 | 0.101 | 0.195 | 0 |

Table S9. Regularised partial correlations weights for girls in the 6-8 years old group.

|  | Ls1 | Ls2 | Ls3 | Ls4 | Ls5 | Ls6 | Bs1 | Bs2 | Bs3 | Bs4 | Bs5 | Bs6 | Bs7 |
| --- | --- | --- | --- | --- | --- | --- | --- | --- | --- | --- | --- | --- | --- |
| Ls1 | 0 | 0.133 | 0.13 | 0.072 | 0.115 | 0 | 0.059 | 0.064 | 0.045 | 0.009 | 0.092 | 0.056 | 0 |
| Ls2 | 0.133 | 0 | 0.091 | 0.105 | 0.067 | 0 | 0.062 | 0.021 | 0.095 | 0.022 | 0.042 | 0 | 0.022 |
| Ls3 | 0.13 | 0.091 | 0 | 0.116 | 0.137 | 0.16 | 0.021 | 0.016 | 0.001 | 0.045 | 0 | 0.026 | 0.031 |
| Ls4 | 0.072 | 0.105 | 0.116 | 0 | 0.109 | 0.108 | 0.035 | 0 | 0.032 | 0.058 | 0 | 0.061 | 0.062 |
| Ls5 | 0.115 | 0.067 | 0.137 | 0.109 | 0 | 0.223 | 0.015 | 0.045 | 0.027 | 0.033 | 0 | 0.034 | 0 |
| Ls6 | 0 | 0 | 0.16 | 0.108 | 0.223 | 0 | -0.023 | 0 | 0 | 0.103 | -0.01 | -0.023 | -0.024 |
| Bs1 | 0.059 | 0.062 | 0.021 | 0.035 | 0.015 | -0.023 | 0 | 0.181 | 0.031 | 0.027 | 0.084 | 0.087 | 0.102 |
| Bs2 | 0.064 | 0.021 | 0.016 | 0 | 0.045 | 0 | 0.181 | 0 | 0.158 | 0.059 | 0.125 | 0.051 | 0.108 |
| Bs3 | 0.045 | 0.095 | 0.001 | 0.032 | 0.027 | 0 | 0.031 | 0.158 | 0 | 0.066 | 0.12 | 0 | 0.05 |
| Bs4 | 0.009 | 0.022 | 0.045 | 0.058 | 0.033 | 0.103 | 0.027 | 0.059 | 0.066 | 0 | 0.038 | 0.072 | 0.102 |
| Bs5 | 0.092 | 0.042 | 0 | 0 | 0 | -0.01 | 0.084 | 0.125 | 0.12 | 0.038 | 0 | 0.091 | 0.055 |
| Bs6 | 0.056 | 0 | 0.026 | 0.061 | 0.034 | -0.023 | 0.087 | 0.051 | 0 | 0.072 | 0.091 | 0 | 0.221 |
| Bs7 | 0 | 0.022 | 0.031 | 0.062 | 0 | -0.024 | 0.102 | 0.108 | 0.05 | 0.102 | 0.055 | 0.221 | 0 |

Table S10. Regularised partial correlations weights for girls in the 9-11 years old group.

|  | Ls1 | Ls2 | Ls3 | Ls4 | Ls5 | Ls6 | Bs1 | Bs2 | Bs3 | Bs4 | Bs5 | Bs6 | Bs7 |
| --- | --- | --- | --- | --- | --- | --- | --- | --- | --- | --- | --- | --- | --- |
| Ls1 | 0 | 0.089 | 0.168 | 0.053 | 0.088 | 0 | 0.052 | 0.057 | 0.03 | 0 | 0.085 | 0.062 | 0.026 |
| Ls2 | 0.089 | 0 | 0.096 | 0.107 | 0.02 | 0 | 0.031 | 0.064 | 0.039 | 0.024 | 0.04 | 0.006 | 0 |
| Ls3 | 0.168 | 0.096 | 0 | 0.064 | 0.166 | 0.101 | 0 | 0 | 0 | 0.069 | 0.045 | 0.038 | 0.011 |
| Ls4 | 0.053 | 0.107 | 0.064 | 0 | 0.17 | 0.096 | 0.01 | 0.016 | 0.024 | 0.064 | 0.011 | 0.036 | 0.063 |
| Ls5 | 0.088 | 0.02 | 0.166 | 0.17 | 0 | 0.222 | 0 | 0.031 | 0.021 | 0 | 0.038 | 0.035 | 0 |
| Ls6 | 0 | 0 | 0.101 | 0.096 | 0.222 | 0 | 0.006 | 0 | -0.079 | 0.08 | 0 | 0.013 | 0 |
| Bs1 | 0.052 | 0.031 | 0 | 0.01 | 0 | 0.006 | 0 | 0.229 | 0.013 | 0.026 | 0.055 | 0.102 | 0.106 |
| Bs2 | 0.057 | 0.064 | 0 | 0.016 | 0.031 | 0 | 0.229 | 0 | 0.147 | 0 | 0.04 | 0.099 | 0.072 |
| Bs3 | 0.03 | 0.039 | 0 | 0.024 | 0.021 | -0.079 | 0.013 | 0.147 | 0 | 0.033 | 0.155 | 0 | 0.097 |
| Bs4 | 0 | 0.024 | 0.069 | 0.064 | 0 | 0.08 | 0.026 | 0 | 0.033 | 0 | 0.04 | 0.056 | 0.084 |
| Bs5 | 0.085 | 0.04 | 0.045 | 0.011 | 0.038 | 0 | 0.055 | 0.04 | 0.155 | 0.04 | 0 | 0.098 | 0 |
| Bs6 | 0.062 | 0.006 | 0.038 | 0.036 | 0.035 | 0.013 | 0.102 | 0.099 | 0 | 0.056 | 0.098 | 0 | 0.283 |
| Bs7 | 0.026 | 0 | 0.011 | 0.063 | 0 | 0 | 0.106 | 0.072 | 0.097 | 0.084 | 0 | 0.283 | 0 |

Table S11. Frequencies for boys and girls across age groups.

| Age strata | Boys | Girls |
| --- | --- | --- |
| 3-5 | 1780 | 1745 |
| 6-8 | 4021 | 3861 |
| 9-11 | 2944 | 2638 |

Table S12. Partial correlations for the all-sample.

|  | Ls1 | Ls2 | Ls3 | Ls4 | Ls5 | Ls6 | Bs1 | Bs2 | Bs3 | Bs4 | Bs5 | Bs6 | Bs7 |
| --- | --- | --- | --- | --- | --- | --- | --- | --- | --- | --- | --- | --- | --- |
| Ls1 | 1 | 0.16 | 0.148 | 0.073 | 0.115 | 0.092 | 0.09 | 0.047 | 0.038 | 0.071 | 0.06 | 0.067 | 0.034 |
| Ls2 | 0.16 | 1 | 0.108 | 0.136 | 0.063 | 0.06 | 0.066 | 0.035 | 0.099 | 0.043 | -0.004 | 0.029 | 0.026 |
| Ls3 | 0.148 | 0.108 | 1 | 0.144 | 0.166 | 0.177 | 0.037 | 0.034 | 0.059 | 0.089 | 0.029 | 0.042 | 0.031 |
| Ls4 | 0.073 | 0.136 | 0.144 | 1 | 0.132 | 0.137 | 0.009 | 0.007 | 0.07 | 0.063 | -0.005 | 0.039 | 0.052 |
| Ls5 | 0.115 | 0.063 | 0.166 | 0.132 | 1 | 0.181 | 0.018 | 0.039 | 0.038 | 0.022 | 0.013 | 0.032 | 0.011 |
| Ls6 | 0.092 | 0.06 | 0.177 | 0.137 | 0.181 | 1 | 0.022 | 0.001 | -0.017 | 0.172 | 0.024 | -0.027 | -0.015 |
| Bs1 | 0.09 | 0.066 | 0.037 | 0.009 | 0.018 | 0.022 | 1 | 0.21 | 0.05 | 0.063 | 0.1 | 0.126 | 0.109 |
| Bs2 | 0.047 | 0.035 | 0.034 | 0.007 | 0.039 | 0.001 | 0.21 | 1 | 0.216 | 0.046 | 0.171 | 0.081 | 0.115 |
| Bs3 | 0.038 | 0.099 | 0.059 | 0.07 | 0.038 | -0.017 | 0.05 | 0.216 | 1 | 0.088 | 0.178 | 0.012 | 0.069 |
| Bs4 | 0.071 | 0.043 | 0.089 | 0.063 | 0.022 | 0.172 | 0.063 | 0.046 | 0.088 | 1 | 0.077 | 0.046 | 0.134 |
| Bs5 | 0.06 | -0.004 | 0.029 | -0.005 | 0.013 | 0.024 | 0.1 | 0.171 | 0.178 | 0.077 | 1 | 0.1 | 0.102 |
| Bs6 | 0.067 | 0.029 | 0.042 | 0.039 | 0.032 | -0.027 | 0.126 | 0.081 | 0.012 | 0.046 | 0.1 | 1 | 0.252 |
| Bs7 | 0.034 | 0.026 | 0.031 | 0.052 | 0.011 | -0.015 | 0.109 | 0.115 | 0.069 | 0.134 | 0.102 | 0.252 | 1 |

Table S13. Partial correlations for the 3-5 years old group.

|  | Ls1 | Ls2 | Ls3 | Ls4 | Ls5 | Ls6 | Bs1 | Bs2 | Bs3 | Bs4 | Bs5 | Bs6 | Bs7 |
| --- | --- | --- | --- | --- | --- | --- | --- | --- | --- | --- | --- | --- | --- |
| Ls1 | 1 | 0.182 | 0.078 | 0.141 | 0.132 | 0.186 | 0.099 | -0.031 | 0.002 | 0.123 | -0.035 | 0.058 | 0.045 |
| Ls2 | 0.182 | 1 | 0.068 | 0.146 | 0.062 | 0.191 | 0.091 | 0.002 | 0.027 | 0.004 | -0.064 | 0.03 | 0.001 |
| Ls3 | 0.078 | 0.068 | 1 | 0.214 | 0.183 | 0.143 | 0.068 | 0.047 | 0.124 | 0.063 | 0.035 | 0.026 | 0.014 |
| Ls4 | 0.141 | 0.146 | 0.214 | 1 | 0.115 | 0.106 | -0.064 | 0.027 | 0.077 | -0.044 | 0.021 | 0.08 | 0.086 |
| Ls5 | 0.132 | 0.062 | 0.183 | 0.115 | 1 | 0.097 | 0.073 | 0.026 | 0.017 | 0.007 | 0.005 | 0.018 | 0.015 |
| Ls6 | 0.186 | 0.191 | 0.143 | 0.106 | 0.097 | 1 | 0.039 | 0.022 | 0.041 | 0.105 | 0.015 | -0.011 | -0.024 |
| Bs1 | 0.099 | 0.091 | 0.068 | -0.064 | 0.073 | 0.039 | 1 | 0.193 | 0.042 | 0.072 | 0.102 | 0.155 | 0.109 |
| Bs2 | -0.031 | 0.002 | 0.047 | 0.027 | 0.026 | 0.022 | 0.193 | 1 | 0.217 | 0.045 | 0.165 | 0.065 | 0.103 |
| Bs3 | 0.002 | 0.027 | 0.124 | 0.077 | 0.017 | 0.041 | 0.042 | 0.217 | 1 | 0.052 | 0.124 | 0.005 | 0.024 |
| Bs4 | 0.123 | 0.004 | 0.063 | -0.044 | 0.007 | 0.105 | 0.072 | 0.045 | 0.052 | 1 | 0.082 | 0.065 | 0.176 |
| Bs5 | -0.035 | -0.064 | 0.035 | 0.021 | 0.005 | 0.015 | 0.102 | 0.165 | 0.124 | 0.082 | 1 | 0.144 | 0.162 |
| Bs6 | 0.058 | 0.03 | 0.026 | 0.08 | 0.018 | -0.011 | 0.155 | 0.065 | 0.005 | 0.065 | 0.144 | 1 | 0.216 |
| Bs7 | 0.045 | 0.001 | 0.014 | 0.086 | 0.015 | -0.024 | 0.109 | 0.103 | 0.024 | 0.176 | 0.162 | 0.216 | 1 |

Table S14. Partial correlations for the 6-8 years old group.

|  | Ls1 | Ls2 | Ls3 | Ls4 | Ls5 | Ls6 | Bs1 | Bs2 | Bs3 | Bs4 | Bs5 | Bs6 | Bs7 |
| --- | --- | --- | --- | --- | --- | --- | --- | --- | --- | --- | --- | --- | --- |
| Ls1 | 1 | 0.12 | 0.14 | 0.042 | 0.105 | 0.025 | 0.061 | 0.063 | 0.061 | 0.021 | 0.086 | 0.076 | 0.002 |
| Ls2 | 0.12 | 1 | 0.102 | 0.125 | 0.082 | -0.011 | 0.039 | 0.013 | 0.088 | 0.024 | 0.018 | 0.015 | 0.039 |
| Ls3 | 0.14 | 0.102 | 1 | 0.118 | 0.146 | 0.169 | 0.024 | 0.025 | 0.023 | 0.058 | -0.011 | 0.039 | 0.026 |
| Ls4 | 0.042 | 0.125 | 0.118 | 1 | 0.126 | 0.102 | 0.027 | -0.016 | 0.05 | 0.07 | -0.021 | 0.027 | 0.026 |
| Ls5 | 0.105 | 0.082 | 0.146 | 0.126 | 1 | 0.189 | 0.017 | 0.041 | 0.046 | 0.024 | -0.01 | 0.039 | 0.009 |
| Ls6 | 0.025 | -0.011 | 0.169 | 0.102 | 0.189 | 1 | -0.018 | -0.009 | -0.046 | 0.15 | 0.015 | -0.043 | -0.044 |
| Bs1 | 0.061 | 0.039 | 0.024 | 0.027 | 0.017 | -0.018 | 1 | 0.18 | 0.048 | 0.029 | 0.105 | 0.099 | 0.104 |
| Bs2 | 0.063 | 0.013 | 0.025 | -0.016 | 0.041 | -0.009 | 0.18 | 1 | 0.176 | 0.032 | 0.179 | 0.075 | 0.112 |
| Bs3 | 0.061 | 0.088 | 0.023 | 0.05 | 0.046 | -0.046 | 0.048 | 0.176 | 1 | 0.063 | 0.153 | 0.02 | 0.053 |
| Bs4 | 0.021 | 0.024 | 0.058 | 0.07 | 0.024 | 0.15 | 0.029 | 0.032 | 0.063 | 1 | 0.053 | 0.039 | 0.095 |
| Bs5 | 0.086 | 0.018 | -0.011 | -0.021 | -0.01 | 0.015 | 0.105 | 0.179 | 0.153 | 0.053 | 1 | 0.089 | 0.084 |
| Bs6 | 0.076 | 0.015 | 0.039 | 0.027 | 0.039 | -0.043 | 0.099 | 0.075 | 0.02 | 0.039 | 0.089 | 1 | 0.241 |
| Bs7 | 0.002 | 0.039 | 0.026 | 0.026 | 0.009 | -0.044 | 0.104 | 0.112 | 0.053 | 0.095 | 0.084 | 0.241 | 1 |

Table S15. Partial correlations for the 9-11 years old group.

|  | Ls1 | Ls2 | Ls3 | Ls4 | Ls5 | Ls6 | Bs1 | Bs2 | Bs3 | Bs4 | Bs5 | Bs6 | Bs7 |
| --- | --- | --- | --- | --- | --- | --- | --- | --- | --- | --- | --- | --- | --- |
| Ls1 | 1 | 0.09 | 0.141 | 0.038 | 0.124 | 0.011 | 0.064 | 0.072 | 0.046 | 0.013 | 0.102 | 0.034 | 0.051 |
| Ls2 | 0.09 | 1 | 0.098 | 0.098 | 0.032 | -0.024 | 0.04 | 0.055 | 0.057 | 0.025 | -0.002 | 0.055 | -0.011 |
| Ls3 | 0.141 | 0.098 | 1 | 0.067 | 0.173 | 0.122 | -0.017 | -0.002 | 0.036 | 0.067 | 0.047 | 0.054 | 0.012 |
| Ls4 | 0.038 | 0.098 | 0.067 | 1 | 0.144 | 0.135 | -0.005 | -0.001 | 0.031 | 0.057 | -0.024 | 0.032 | 0.04 |
| Ls5 | 0.124 | 0.032 | 0.173 | 0.144 | 1 | 0.223 | -0.024 | 0.04 | 0.01 | 0.019 | 0.052 | 0.035 | 0.008 |
| Ls6 | 0.011 | -0.024 | 0.122 | 0.135 | 0.223 | 1 | 0.021 | -0.039 | -0.075 | 0.104 | 0.028 | -0.015 | 0.008 |
| Bs1 | 0.064 | 0.04 | -0.017 | -0.005 | -0.024 | 0.021 | 1 | 0.243 | 0.034 | 0.034 | 0.073 | 0.143 | 0.087 |
| Bs2 | 0.072 | 0.055 | -0.002 | -0.001 | 0.04 | -0.039 | 0.243 | 1 | 0.129 | -0.006 | 0.111 | 0.103 | 0.108 |
| Bs3 | 0.046 | 0.057 | 0.036 | 0.031 | 0.01 | -0.075 | 0.034 | 0.129 | 1 | 0.026 | 0.174 | 0.016 | 0.097 |
| Bs4 | 0.013 | 0.025 | 0.067 | 0.057 | 0.019 | 0.104 | 0.034 | -0.006 | 0.026 | 1 | 0.035 | 0.053 | 0.056 |
| Bs5 | 0.102 | -0.002 | 0.047 | -0.024 | 0.052 | 0.028 | 0.073 | 0.111 | 0.174 | 0.035 | 1 | 0.084 | 0.036 |
| Bs6 | 0.034 | 0.055 | 0.054 | 0.032 | 0.035 | -0.015 | 0.143 | 0.103 | 0.016 | 0.053 | 0.084 | 1 | 0.301 |
| Bs7 | 0.051 | -0.011 | 0.012 | 0.04 | 0.008 | 0.008 | 0.087 | 0.108 | 0.097 | 0.056 | 0.036 | 0.301 | 1 |

Table S16. Partial correlations for boys in the 3-5 years old group.

|  | Ls1 | Ls2 | Ls3 | Ls4 | Ls5 | Ls6 | Bs1 | Bs2 | Bs3 | Bs4 | Bs5 | Bs6 | Bs7 |
| --- | --- | --- | --- | --- | --- | --- | --- | --- | --- | --- | --- | --- | --- |
| Ls1 | 1 | 0.158 | 0.114 | 0.092 | 0.118 | 0.205 | 0.106 | -0.062 | -0.005 | 0.135 | 0.029 | 0.094 | 0.01 |
| Ls2 | 0.158 | 1 | 0.053 | 0.163 | 0.033 | 0.131 | 0.132 | 0.033 | 0.027 | -0.03 | -0.037 | 0.013 | 0.017 |
| Ls3 | 0.114 | 0.053 | 1 | 0.214 | 0.127 | 0.186 | 0.087 | 0.046 | 0.135 | 0.101 | 0.016 | -0.03 | 0.02 |
| Ls4 | 0.092 | 0.163 | 0.214 | 1 | 0.113 | 0.095 | -0.091 | 0.047 | 0.056 | -0.055 | 0.04 | 0.093 | 0.088 |
| Ls5 | 0.118 | 0.033 | 0.127 | 0.113 | 1 | 0.185 | 0.032 | 0.058 | 0.018 | -0.011 | 0.017 | 0.024 | 0.014 |
| Ls6 | 0.205 | 0.131 | 0.186 | 0.095 | 0.185 | 1 | 0.034 | 0.06 | 0.059 | 0.075 | 0.012 | -0.006 | -0.054 |
| Bs1 | 0.106 | 0.132 | 0.087 | -0.091 | 0.032 | 0.034 | 1 | 0.236 | 0.029 | 0.041 | 0.067 | 0.148 | 0.096 |
| Bs2 | -0.062 | 0.033 | 0.046 | 0.047 | 0.058 | 0.06 | 0.236 | 1 | 0.245 | 0.036 | 0.116 | 0.113 | 0.079 |
| Bs3 | -0.005 | 0.027 | 0.135 | 0.056 | 0.018 | 0.059 | 0.029 | 0.245 | 1 | 0.074 | 0.121 | -0.04 | 0.07 |
| Bs4 | 0.135 | -0.03 | 0.101 | -0.055 | -0.011 | 0.075 | 0.041 | 0.036 | 0.074 | 1 | 0.052 | 0.071 | 0.187 |
| Bs5 | 0.029 | -0.037 | 0.016 | 0.04 | 0.017 | 0.012 | 0.067 | 0.116 | 0.121 | 0.052 | 1 | 0.118 | 0.2 |
| Bs6 | 0.094 | 0.013 | -0.03 | 0.093 | 0.024 | -0.006 | 0.148 | 0.113 | -0.04 | 0.071 | 0.118 | 1 | 0.239 |
| Bs7 | 0.01 | 0.017 | 0.02 | 0.088 | 0.014 | -0.054 | 0.096 | 0.079 | 0.07 | 0.187 | 0.2 | 0.239 | 1 |

Table S17. Partial correlations for boys in the 6-8 years old group.

|  | Ls1 | Ls2 | Ls3 | Ls4 | Ls5 | Ls6 | Bs1 | Bs2 | Bs3 | Bs4 | Bs5 | Bs6 | Bs7 |
| --- | --- | --- | --- | --- | --- | --- | --- | --- | --- | --- | --- | --- | --- |
| Ls1 | 1 | 0.105 | 0.148 | 0.022 | 0.098 | 0.034 | 0.055 | 0.057 | 0.077 | 0.034 | 0.054 | 0.09 | 0.01 |
| Ls2 | 0.105 | 1 | 0.091 | 0.105 | 0.071 | 0.005 | 0.035 | 0.032 | 0.082 | 0.016 | 0.054 | 0.045 | 0.051 |
| Ls3 | 0.148 | 0.091 | 1 | 0.104 | 0.147 | 0.164 | 0.031 | 0.039 | 0.032 | 0.068 | 0.033 | 0.049 | 0.009 |
| Ls4 | 0.022 | 0.105 | 0.104 | 1 | 0.123 | 0.09 | 0.034 | 0.014 | 0.065 | 0.069 | 0.022 | 0.011 | -0.008 |
| Ls5 | 0.098 | 0.071 | 0.147 | 0.123 | 1 | 0.151 | 0.019 | 0.04 | 0.051 | 0.015 | 0.024 | 0.032 | 0.039 |
| Ls6 | 0.034 | 0.005 | 0.164 | 0.09 | 0.151 | 1 | 0.002 | -0.014 | -0.07 | 0.174 | 0.037 | -0.041 | -0.053 |
| Bs1 | 0.055 | 0.035 | 0.031 | 0.034 | 0.019 | 0.002 | 1 | 0.153 | 0.064 | 0.032 | 0.069 | 0.101 | 0.1 |
| Bs2 | 0.057 | 0.032 | 0.039 | 0.014 | 0.04 | -0.014 | 0.153 | 1 | 0.18 | 0.013 | 0.145 | 0.081 | 0.106 |
| Bs3 | 0.077 | 0.082 | 0.032 | 0.065 | 0.051 | -0.07 | 0.064 | 0.18 | 1 | 0.058 | 0.162 | 0.038 | 0.05 |
| Bs4 | 0.034 | 0.016 | 0.068 | 0.069 | 0.015 | 0.174 | 0.032 | 0.013 | 0.058 | 1 | 0.076 | 0.01 | 0.082 |
| Bs5 | 0.054 | 0.054 | 0.033 | 0.022 | 0.024 | 0.037 | 0.069 | 0.145 | 0.162 | 0.076 | 1 | 0.047 | 0.1 |
| Bs6 | 0.09 | 0.045 | 0.049 | 0.011 | 0.032 | -0.041 | 0.101 | 0.081 | 0.038 | 0.01 | 0.047 | 1 | 0.249 |
| Bs7 | 0.01 | 0.051 | 0.009 | -0.008 | 0.039 | -0.053 | 0.1 | 0.106 | 0.05 | 0.082 | 0.1 | 0.249 | 1 |

Table S18. Partial correlations for boys in the 9-11 years old group.

|  | Ls1 | Ls2 | Ls3 | Ls4 | Ls5 | Ls6 | Bs1 | Bs2 | Bs3 | Bs4 | Bs5 | Bs6 | Bs7 |
| --- | --- | --- | --- | --- | --- | --- | --- | --- | --- | --- | --- | --- | --- |
| Ls1 | 1 | 0.082 | 0.108 | 0.021 | 0.143 | 0.029 | 0.071 | 0.087 | 0.064 | 0.029 | 0.117 | 0.013 | 0.067 |
| Ls2 | 0.082 | 1 | 0.088 | 0.069 | 0.03 | -0.033 | 0.052 | 0.06 | 0.071 | 0.011 | 0.022 | 0.102 | -0.009 |
| Ls3 | 0.108 | 0.088 | 1 | 0.063 | 0.177 | 0.13 | -0.025 | 0.004 | 0.069 | 0.058 | 0.056 | 0.069 | 0.004 |
| Ls4 | 0.021 | 0.069 | 0.063 | 1 | 0.121 | 0.153 | -0.005 | 0.003 | 0.035 | 0.038 | 0.014 | 0.048 | 0.016 |
| Ls5 | 0.143 | 0.03 | 0.177 | 0.121 | 1 | 0.208 | -0.016 | 0.034 | -0.011 | 0.042 | 0.075 | 0.038 | 0.011 |
| Ls6 | 0.029 | -0.033 | 0.13 | 0.153 | 0.208 | 1 | 0.014 | -0.056 | -0.052 | 0.112 | 0.056 | -0.048 | 0.036 |
| Bs1 | 0.071 | 0.052 | -0.025 | -0.005 | -0.016 | 0.014 | 1 | 0.233 | 0.048 | 0.044 | 0.034 | 0.166 | 0.061 |
| Bs2 | 0.087 | 0.06 | 0.004 | 0.003 | 0.034 | -0.056 | 0.233 | 1 | 0.101 | -0.001 | 0.113 | 0.092 | 0.137 |
| Bs3 | 0.064 | 0.071 | 0.069 | 0.035 | -0.011 | -0.052 | 0.048 | 0.101 | 1 | 0.012 | 0.159 | 0.036 | 0.089 |
| Bs4 | 0.029 | 0.011 | 0.058 | 0.038 | 0.042 | 0.112 | 0.044 | -0.001 | 0.012 | 1 | 0.052 | 0.059 | 0.019 |
| Bs5 | 0.117 | 0.022 | 0.056 | 0.014 | 0.075 | 0.056 | 0.034 | 0.113 | 0.159 | 0.052 | 1 | 0.015 | 0.069 |
| Bs6 | 0.013 | 0.102 | 0.069 | 0.048 | 0.038 | -0.048 | 0.166 | 0.092 | 0.036 | 0.059 | 0.015 | 1 | 0.311 |
| Bs7 | 0.067 | -0.009 | 0.004 | 0.016 | 0.011 | 0.036 | 0.061 | 0.137 | 0.089 | 0.019 | 0.069 | 0.311 | 1 |

Table S19. Partial correlations for boys in the 3-5 years old group.

|  | Ls1 | Ls2 | Ls3 | Ls4 | Ls5 | Ls6 | Bs1 | Bs2 | Bs3 | Bs4 | Bs5 | Bs6 | Bs7 |
| --- | --- | --- | --- | --- | --- | --- | --- | --- | --- | --- | --- | --- | --- |
| Ls1 | 1 | 0.214 | 0.057 | 0.205 | 0.139 | 0.161 | 0.082 | -0.017 | -0.001 | 0.114 | -0.123 | 0.028 | 0.071 |
| Ls2 | 0.214 | 1 | 0.079 | 0.107 | 0.099 | 0.249 | 0.042 | -0.014 | 0.033 | 0.038 | -0.067 | 0.047 | -0.019 |
| Ls3 | 0.057 | 0.079 | 1 | 0.193 | 0.23 | 0.106 | 0.054 | 0.084 | 0.113 | 0.013 | 0.08 | 0.082 | 0.018 |
| Ls4 | 0.205 | 0.107 | 0.193 | 1 | 0.111 | 0.115 | -0.021 | 0.046 | 0.113 | -0.043 | 0.037 | 0.064 | 0.082 |
| Ls5 | 0.139 | 0.099 | 0.23 | 0.111 | 1 | 0.001 | 0.122 | -0.016 | 0.012 | 0.029 | -0.007 | 0.004 | 0.022 |
| Ls6 | 0.161 | 0.249 | 0.106 | 0.115 | 0.001 | 1 | 0.052 | -0.03 | 0.017 | 0.129 | 0.03 | -0.016 | 0.009 |
| Bs1 | 0.082 | 0.042 | 0.054 | -0.021 | 0.122 | 0.052 | 1 | 0.109 | 0.049 | 0.11 | 0.12 | 0.158 | 0.124 |
| Bs2 | -0.017 | -0.014 | 0.084 | 0.046 | -0.016 | -0.03 | 0.109 | 1 | 0.142 | 0.084 | 0.172 | 0.009 | 0.123 |
| Bs3 | -0.001 | 0.033 | 0.113 | 0.113 | 0.012 | 0.017 | 0.049 | 0.142 | 1 | 0.035 | 0.107 | 0.056 | -0.028 |
| Bs4 | 0.114 | 0.038 | 0.013 | -0.043 | 0.029 | 0.129 | 0.11 | 0.084 | 0.035 | 1 | 0.115 | 0.062 | 0.16 |
| Bs5 | -0.123 | -0.067 | 0.08 | 0.037 | -0.007 | 0.03 | 0.12 | 0.172 | 0.107 | 0.115 | 1 | 0.161 | 0.108 |
| Bs6 | 0.028 | 0.047 | 0.082 | 0.064 | 0.004 | -0.016 | 0.158 | 0.009 | 0.056 | 0.062 | 0.161 | 1 | 0.199 |
| Bs7 | 0.071 | -0.019 | 0.018 | 0.082 | 0.022 | 0.009 | 0.124 | 0.123 | -0.028 | 0.16 | 0.108 | 0.199 | 1 |

Table S20. Partial correlations for girls in the 6-8 years old group.

|  | Ls1 | Ls2 | Ls3 | Ls4 | Ls5 | Ls6 | Bs1 | Bs2 | Bs3 | Bs4 | Bs5 | Bs6 | Bs7 |
| --- | --- | --- | --- | --- | --- | --- | --- | --- | --- | --- | --- | --- | --- |
| Ls1 | 1 | 0.136 | 0.133 | 0.072 | 0.115 | 0.014 | 0.061 | 0.065 | 0.046 | 0.008 | 0.102 | 0.06 | -0.006 |
| Ls2 | 0.136 | 1 | 0.098 | 0.111 | 0.076 | -0.025 | 0.063 | 0.022 | 0.097 | 0.026 | 0.048 | -0.007 | 0.027 |
| Ls3 | 0.133 | 0.098 | 1 | 0.115 | 0.136 | 0.173 | 0.027 | 0.022 | 0.009 | 0.043 | -0.024 | 0.033 | 0.042 |
| Ls4 | 0.072 | 0.111 | 0.115 | 1 | 0.11 | 0.123 | 0.041 | -0.013 | 0.037 | 0.057 | 0.01 | 0.065 | 0.072 |
| Ls5 | 0.115 | 0.076 | 0.136 | 0.11 | 1 | 0.236 | 0.026 | 0.055 | 0.037 | 0.033 | -0.011 | 0.05 | -0.025 |
| Ls6 | 0.014 | -0.025 | 0.173 | 0.123 | 0.236 | 1 | -0.042 | -0.007 | -0.021 | 0.123 | -0.016 | -0.046 | -0.034 |
| Bs1 | 0.061 | 0.063 | 0.027 | 0.041 | 0.026 | -0.042 | 1 | 0.184 | 0.032 | 0.031 | 0.087 | 0.087 | 0.104 |
| Bs2 | 0.065 | 0.022 | 0.022 | -0.013 | 0.055 | -0.007 | 0.184 | 1 | 0.161 | 0.062 | 0.129 | 0.052 | 0.113 |
| Bs3 | 0.046 | 0.097 | 0.009 | 0.037 | 0.037 | -0.021 | 0.032 | 0.161 | 1 | 0.071 | 0.124 | -0.002 | 0.053 |
| Bs4 | 0.008 | 0.026 | 0.043 | 0.057 | 0.033 | 0.123 | 0.031 | 0.062 | 0.071 | 1 | 0.044 | 0.077 | 0.109 |
| Bs5 | 0.102 | 0.048 | -0.024 | 0.01 | -0.011 | -0.016 | 0.087 | 0.129 | 0.124 | 0.044 | 1 | 0.095 | 0.058 |
| Bs6 | 0.06 | -0.007 | 0.033 | 0.065 | 0.05 | -0.046 | 0.087 | 0.052 | -0.002 | 0.077 | 0.095 | 1 | 0.226 |
| Bs7 | -0.006 | 0.027 | 0.042 | 0.072 | -0.025 | -0.034 | 0.104 | 0.113 | 0.053 | 0.109 | 0.058 | 0.226 | 1 |

Table S21. Partial correlations for girls in the 9-11 years old group.

|  | Ls1 | Ls2 | Ls3 | Ls4 | Ls5 | Ls6 | Bs1 | Bs2 | Bs3 | Bs4 | Bs5 | Bs6 | Bs7 |
| --- | --- | --- | --- | --- | --- | --- | --- | --- | --- | --- | --- | --- | --- |
| Ls1 | 1 | 0.093 | 0.175 | 0.056 | 0.095 | -0.014 | 0.06 | 0.058 | 0.032 | -0.009 | 0.088 | 0.063 | 0.029 |
| Ls2 | 0.093 | 1 | 0.101 | 0.112 | 0.026 | -0.009 | 0.037 | 0.069 | 0.044 | 0.032 | 0.043 | 0.011 | -0.014 |
| Ls3 | 0.175 | 0.101 | 1 | 0.063 | 0.168 | 0.11 | -0.006 | -0.003 | 0.003 | 0.076 | 0.048 | 0.038 | 0.018 |
| Ls4 | 0.056 | 0.112 | 0.063 | 1 | 0.172 | 0.106 | 0.015 | 0.019 | 0.029 | 0.069 | 0.012 | 0.033 | 0.07 |
| Ls5 | 0.095 | 0.026 | 0.168 | 0.172 | 1 | 0.235 | -0.03 | 0.047 | 0.032 | -0.013 | 0.043 | 0.038 | 0.004 |
| Ls6 | -0.014 | -0.009 | 0.11 | 0.106 | 0.235 | 1 | 0.033 | -0.024 | -0.1 | 0.094 | 0 | 0.028 | -0.022 |
| Bs1 | 0.06 | 0.037 | -0.006 | 0.015 | -0.03 | 0.033 | 1 | 0.236 | 0.019 | 0.03 | 0.059 | 0.105 | 0.11 |
| Bs2 | 0.058 | 0.069 | -0.003 | 0.019 | 0.047 | -0.024 | 0.236 | 1 | 0.149 | -0.001 | 0.042 | 0.102 | 0.074 |
| Bs3 | 0.032 | 0.044 | 0.003 | 0.029 | 0.032 | -0.1 | 0.019 | 0.149 | 1 | 0.043 | 0.162 | -0.004 | 0.101 |
| Bs4 | -0.009 | 0.032 | 0.076 | 0.069 | -0.013 | 0.094 | 0.03 | -0.001 | 0.043 | 1 | 0.046 | 0.058 | 0.091 |
| Bs5 | 0.088 | 0.043 | 0.048 | 0.012 | 0.043 | 0 | 0.059 | 0.042 | 0.162 | 0.046 | 1 | 0.102 | -0.003 |
| Bs6 | 0.063 | 0.011 | 0.038 | 0.033 | 0.038 | 0.028 | 0.105 | 0.102 | -0.004 | 0.058 | 0.102 | 1 | 0.29 |
| Bs7 | 0.029 | -0.014 | 0.018 | 0.07 | 0.004 | -0.022 | 0.11 | 0.074 | 0.101 | 0.091 | -0.003 | 0.29 | 1 |
